# Dynamic YAP expression in the non-parenchymal liver cell compartment controls heterologous cell communication

**DOI:** 10.1007/s00018-024-05126-1

**Published:** 2024-03-04

**Authors:** Kaijing Liu, Lilija Wehling, Shan Wan, Sofia M. E. Weiler, Marcell Tóth, David Ibberson, Silke Marhenke, Adnan Ali, Macrina Lam, Te Guo, Federico Pinna, Fabiola Pedrini, Amruta Damle-Vartak, Anne Dropmann, Fabian Rose, Silvia Colucci, Wenxiang Cheng, Michaela Bissinger, Jennifer Schmitt, Patrizia Birner, Tanja Poth, Peter Angel, Steven Dooley, Martina U. Muckenthaler, Thomas Longerich, Arndt Vogel, Mathias Heikenwälder, Peter Schirmacher, Kai Breuhahn

**Affiliations:** 1 Department of Medical Oncology, Sun Yat-Sen University Cancer Center, Guangdong, China; 2 Institute of Pathology, University Hospital Heidelberg, Im Neuenheimer Feld 224, 69120 Heidelberg, Germany; 3 Department of Modeling of Biological Processes, COS Heidelberg/BioQuant, Heidelberg University, Heidelberg, Germany; 4 State Key Laboratory of Oncology in South China, Sun Yat-Sen University Cancer Center, Sun Yat-Sen University, Guangzhou, China; 5 Department of Pathology, School of Biology & Basic Medical Sciences, Soochow University, Suzhou, China; 6 Deep Sequencing Core Facility, CellNetworks Excellence Cluster, Heidelberg University, Heidelberg, Germany; 7 Department of Gastroenterology, Hepatology and Endocrinology, Hannover Medical School (MHH), Hannover, Germany; 8 Division of Chronic Inflammation and Cancer, German Cancer Research Center (DKFZ), Heidelberg, Germany; 9 Division of Signal Transduction and Growth Control, German Cancer Research Center (DKFZ), Heidelberg, Germany; 10 Department of Medicine II, Molecular Hepatology Section, Medical Faculty Mannheim, Heidelberg University, Mannheim, Germany; 11 Department of Pediatric Oncology, Hematology & Immunology, University Hospital Heidelberg, Heidelberg, Germany; 12 Translational Medicine R&D Center, Institute of Biomedical & Health Engineering, Shenzhen Institutes of Advanced Technology, Chinese Academy of Sciences, Shenzhen, China; 13 Present Address: European Molecular Biology Laboratory (EMBL), Heidelberg, Germany

**Keywords:** Hippo pathway, TAZ, Liver damage, Kupffer cell, Endothelial cell, Hepatocyte, Cholangiocyte, Proteomics, Single-cell analysis

## Abstract

**Introduction:**

The Hippo pathway and its transcriptional effectors yes-associated protein (YAP) and transcriptional coactivator with PDZ-binding motif (TAZ) are targets for cancer therapy. It is important to determine if the activation of one factor compensates for the inhibition of the other. Moreover, it is unknown if YAP/TAZ-directed perturbation affects cell–cell communication of non-malignant liver cells.

**Materials and Methods:**

To investigate liver-specific phenotypes caused by YAP and TAZ inactivation, we generated mice with hepatocyte (HC) and biliary epithelial cell (BEC)-specific deletions for both factors (YAPKO, TAZKO and double knock-out (DKO)). Immunohistochemistry, single-cell sequencing, and proteomics were used to analyze liver tissues and serum.

**Results:**

The loss of BECs, liver fibrosis, and necrosis characterized livers from YAPKO and DKO mice. This phenotype was weakened in DKO tissues compared to specimens from YAPKO animals. After depletion of YAP in HCs and BECs, YAP expression was induced in non-parenchymal cells (NPCs) in a cholestasis-independent manner. YAP positivity was detected in subgroups of Kupffer cells (KCs) and endothelial cells (ECs). The secretion of pro-inflammatory chemokines and cytokines such as C-X-C motif chemokine ligand 11 (CXCL11), fms-related receptor tyrosine kinase 3 ligand (FLT3L), and soluble intercellular adhesion molecule-1 (ICAM1) was increased in the serum of YAPKO animals. YAP activation in NPCs could contribute to inflammation via TEA domain transcription factor (TEAD)-dependent transcriptional regulation of secreted factors.

**Conclusion:**

YAP inactivation in HCs and BECs causes liver damage, and concomitant TAZ deletion does not enhance but reduces this phenotype. Additionally, we present a new mechanism by which YAP contributes to cell–cell communication originating from NPCs.

**Supplementary Information:**

The online version contains supplementary material available at 10.1007/s00018-024-05126-1.

## Introduction

Regulated by cell–cell contact, extracellular matrix stiffness, and cell polarity, the Hippo pathway integrates spatial information into a cellular response [1]. For this, a core serine/threonine kinase cassette consisting of mammalian STE20-like protein kinase (MST)-1/2 and large tumor suppressor kinase (LATS) 1/2 negatively regulates the phosphorylation and subcellular localization of the transcriptional co-activators yes-associated protein (YAP) and transcriptional coactivator with PDZ-binding motif (TAZ). Nuclear accumulation of YAP/TAZ and binding to transcription factors with DNA-binding capacity such as TEA domain transcription factor (TEAD) family members and forkhead box M1 (FOXM1) controls the expression of target genes involved in cell differentiation and proliferation [2–4].

Although YAP and TAZ bind similar promoter regions and regulate similar sets of target genes, it is questionable if they facilitate identical cellular functions [5–7]. For example, YAP-knock-out mice die during embryogenesis with defects in yolk sac vasculogenesis, chorioallantoic fusion, and body axis elongation. In contrast, TAZ-null mice are viable but predisposed to cystic kidney disease and lung emphysema [8, 9]. Indeed, several studies demonstrated that YAP and TAZ are not identical but also induce specific cellular processes during regeneration, and tumorigenesis in different organs such as the liver and lung [10, 11]. However, there is currently no systematic comparison of the cell type-specific dynamic expression of both factors in vivo.

As extensively examined for the liver, dysregulation of Hippo pathway constituents leads to hepatomegaly and tumor formation, as exemplified by MST1/2 deletions or overexpression of YAP in hepatocytes (HCs) [4, 12, 13]. Although comparable data for TAZ overexpression do not exist, hydrodynamic gene delivery experiments targeting HCs revealed oncogenic properties of TAZ [14, 15]. Strategies for targeting YAP and TAZ due to their central role in tumorigenesis are of broad and current interest. Verteporfin, a drug that disrupts the binding of YAP/TAZ to TEAD transcription factors [16, 17], and more recently, small molecule inhibitors of TEAD palmitoylation with in vivo efficiency have been published [18–20]. However, it is unknown if the combined inactivation of YAP and TAZ may cause adverse effects such as hepatotoxicity. In addition, inhibitory drugs that efficiently perturb YAP and TAZ may impair liver functionality by affecting non-tumorous cells. This could point to the necessity to develop YAP- or TAZ-specific inhibitors, which would in part preserve the biological properties of the Hippo pathway.

In this context, liver-specific genetic silencing of YAP or YAP/TAZ causes severe developmental defects of the biliary tree followed by cholestasis, inflammation, and hepatocellular damage [3, 21, 22]. Moreover, first results point to additive or synergistic effects in YAP/TAZ double knock-out mice, suggesting that both proteins are important for bile duct formation and function [3]. These findings suggest that cancer therapies targeting YAP/TAZ may affect both malignant and non-malignant liver cells. Therefore, pharmacological YAP/TAZ perturbation may compromise the biological properties of biliary epithelial cells (BECs; cholangiocytes) as well as non-parenchymal cells (NPCs), including endothelial cells (ECs) of the vascular niche or liver-resident immune cells (Kupffer cells, KCs). However, a comprehensive comparison of how YAP and TAZ expression changes in non-tumorous liver cells under perturbation conditions is missing. Moreover, it is important to understand whether the inactivation of YAP and TAZ affects communication between liver-resident cells of different types.

This study systematically compares cellular and molecular characteristics of liver cells in mice lacking YAP and/or TAZ in HCs and BECs. YAP deficiency-associated loss of BECs caused fibrosis and necrosis, while concomitant TAZ deletion did not enhance but moderately reduced liver damage. Interestingly, the inactivation of YAP in HCs/BECs resulted in YAP induction in EC and KCs subgroups with distinct molecular characteristics. This phenotype was based on heterologous cell–cell communication, as cholestasis did not lead to unspecific YAP activation in NPCs. Chemokines (i.e., C-X-C motif chemokine ligand 11 (CXCL11), fms-related receptor tyrosine kinase 3 ligand (FLT3L)), and surface glycoproteins (i.e., intercellular adhesion molecule-1; ICAM1) that contain TEAD-binding sites in gene promoters accumulate in the blood of YAP-deficient mice. Thus, YAP expression in subgroups of NPCs may contribute to a paracrine communication network in the liver upon its inactivation in HCs/BECs.

## Results

### YAP and TAZ expression in healthy and regenerative liver tissue

**Fig. 1:**
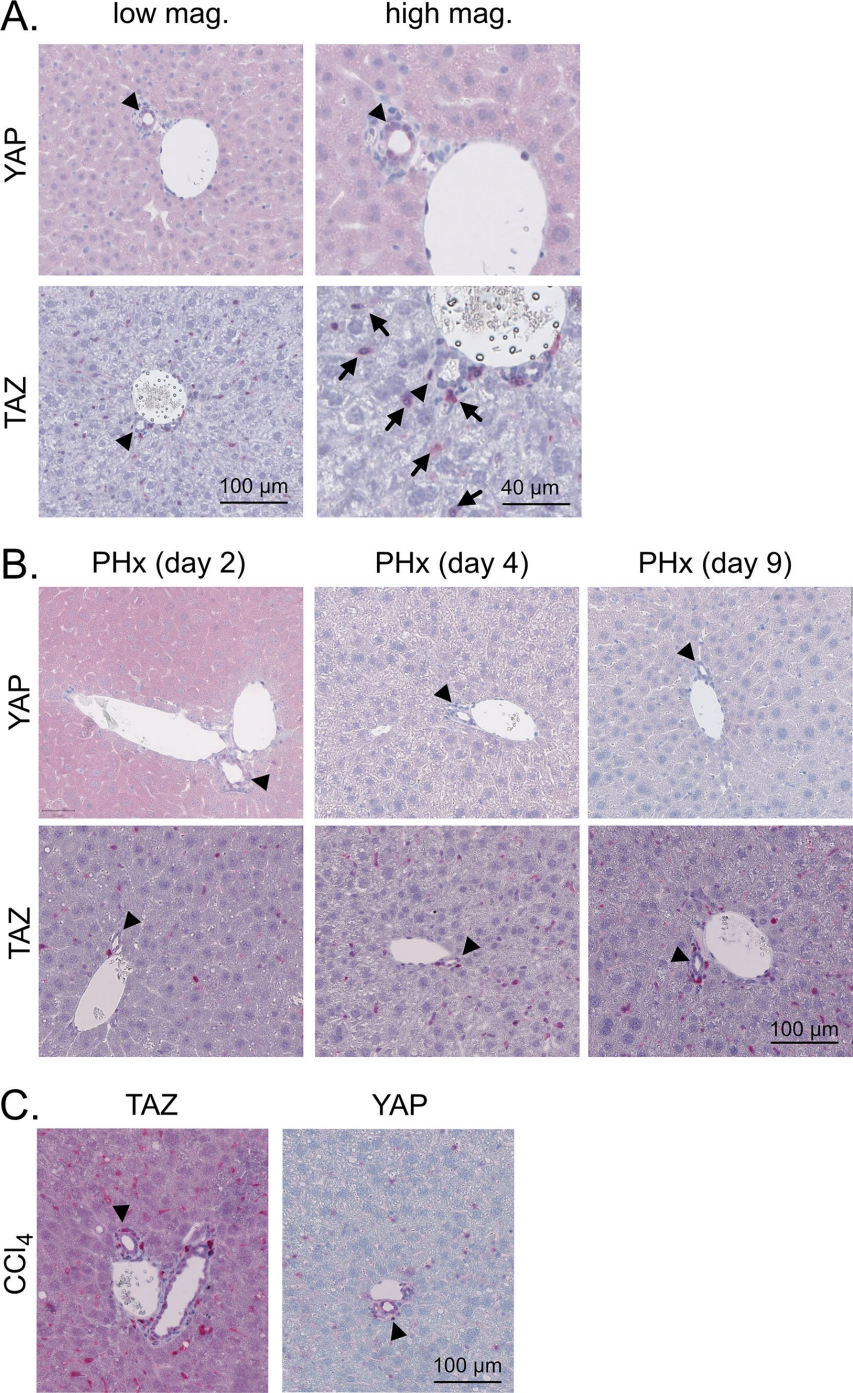
Hepatic expression of YAP and TAZ in healthy, regenerating, and fibrotic liver tissues. **A** Immunohistochemical stains of murine YAP and TAZ in healthy liver tissues of 10-week-old mice. Arrowheads: BECs/bile ducts, arrows: sinusoidal cells. **B** Staining of YAP and TAZ after 70% PHx. Samples were collected at different stages of hepatic regeneration: 2 days (proliferation), 4 days (reorganization), and 9 days (termination). Arrowheads: BECs/bile ducts. **C** Immunohistochemical stains of YAP and TAZ in CCl_4_-induced liver fibrosis. Ten-week-old mice were treated with CCl_4_ for six weeks followed by four weeks without injections. Arrowheads: BECs/bile ducts

To investigate the expression of YAP and TAZ in liver cells comparatively, healthy livers of 10-week-old mice were immunohistochemically stained for both proteins. As expected, YAP was predominantly detected in hepatic BECs but was weakly expressed in parenchymal HCs. No YAP positivity was observed in NPCs such as KCs, hepatic stellate cells (HSCs), and ECs (Fig. 1A). TAZ was not detected in BECs or HCs; however, positive staining was observed in sinusoidal cells, which likely represented KCs, HSCs, or infiltrating immune cells (Fig. 1A).

Previous data have illustrated that YAP and TAZ contribute to physiological liver regeneration, e.g., after partial hepatectomy (PHx) [23, 24]. To test if the cellular expression pattern of both factors changed under regenerative conditions without severe inflammation, we performed 70% PHx. Samples were collected 2, 4, and 9 days post-surgery to cover the proliferation, reorganization, and termination phases, respectively. In the proliferation phase (2 days), moderately elevated YAP expression was observed in the cytoplasm of HCs, but not later or in NPCs. During all stages of liver regeneration, the expression of TAZ was not significantly altered (Fig. 1B).

We then investigated if the cell type-specific expression of YAP and TAZ changed during regenerative processes associated with inflammation. We used a carbon tetrachloride (CCl_4_) protocol, which induced moderate fibrosis characterized by septa connecting the portal tracts (Suppl. Figure S1A). Immunohistochemical stains revealed that YAP was induced in BECs and a few sinusoidal cells; however, a prominent induction in HCs was not observed (Fig. 1C). In contrast, TAZ expression was induced in HCs and sinusoidal cells.

These data illustrate that YAP and TAZ have different expression patterns in parenchymal and non-parenchymal liver cells under steady state, regenerative, and pro-fibrotic conditions.

### The loss of TAZ expression counteracts the effects of YAP depletion

In HCs and BECs with no apparent TAZ positivity, minor TAZ amounts may functionally contribute to cellular processes. In addition, the biological impact of TAZ might be more pronounced in the case of simultaneous YAP silencing. As previous studies showed that YAP and TAZ were dispensable in unchallenged HCs but were critical for bile duct integrity [3, 22], we asked if and to which extent YAP and TAZ synergize in HCs and BECs.

**Fig. 2:**
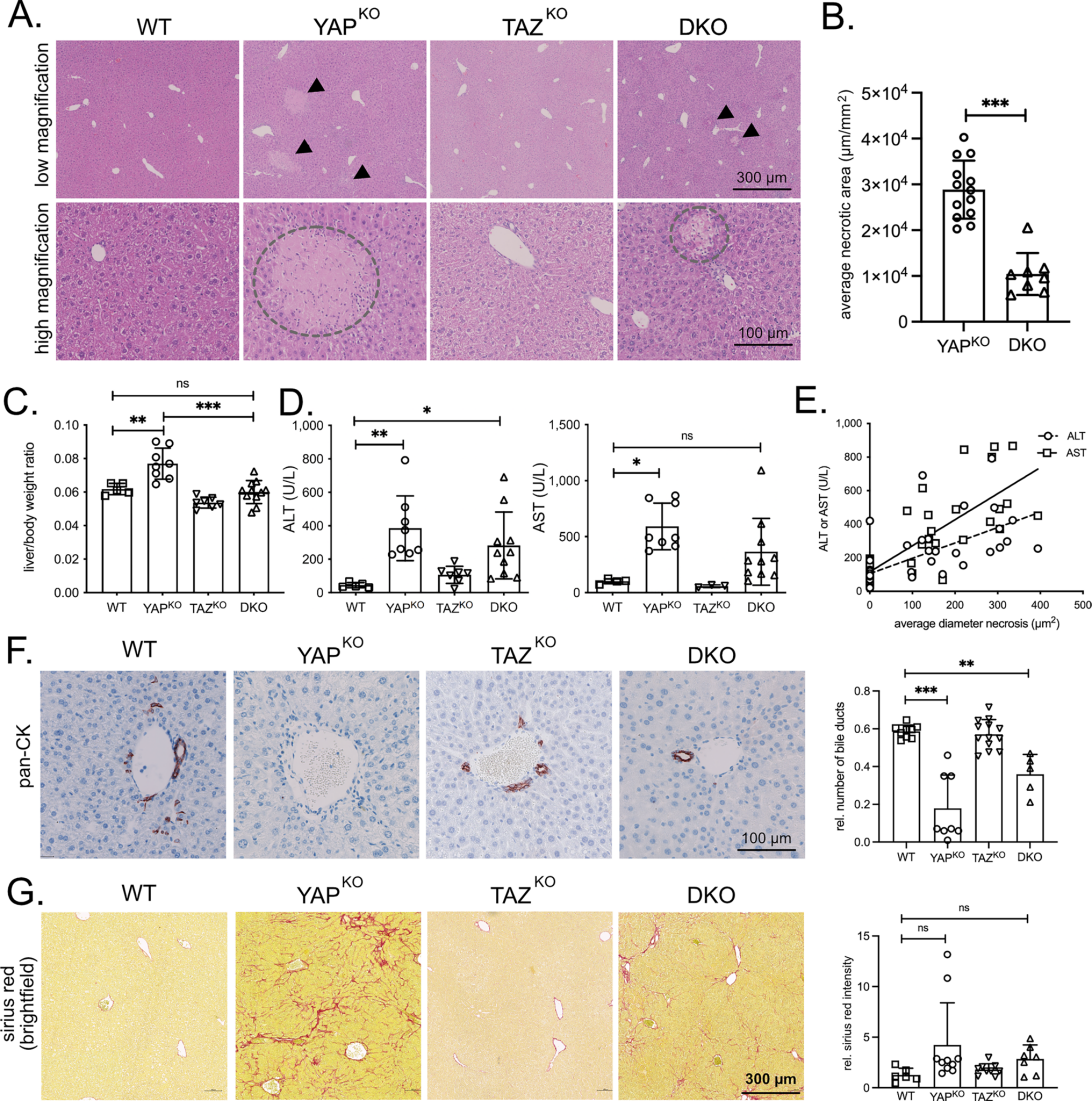
TAZ deficiency diminishes the phenotype caused by YAP silencing.** A** H&E stains of liver tissues from 10 weeks old WT, YAP^KO^, TAZ^KO^, and DKO mice. High and low magnifications are shown. Arrowheads point to necrotic areas in low-magnification panels. Dashed circles indicate liver necrosis at high magnification. **B** Average necrotic area detected in liver specimens from YAP^KO^ (*n* = 13) and DKO (*n* = 8) animals. Mann–Whitney *U* test. ***p ≤ 0.001. **C** Liver/body weight ratio of WT (*n* = 5), YAP^KO^ (*n* = 8), TAZ^KO^ (*n* = 7), and DKO (*n* = 11) male mice. Statistical test: ANOVA with Dunnett’s multiple comparisons test. **p ≤ 0.01, ***p ≤ 0.001, ns: not significant. **D** Serum liver damage markers ALT and AST from male WT (*n* = 5), YAP^KO^ (*n* = 8), TAZ^KO^ (*n* = 7), and DKO (*n* = 10) animals. Statistical test: ANOVA with Dunnett’s multiple comparisons test. *p ≤ 0.05, **p ≤ 0.01, *ns* not significant. **E** Association between average necrosis area and concentration of serum ALT/AST liver damage markers in WT, YAP^KO^, TAZ^KO^, and DKO animals (*n* = 41). r_ALT_: 0.6272; *r*_AST_: 0.7756. Statistical test: Pearson correlation analysis. ***p ≤ 0.001 for ALT and AST. **F** Exemplary immunohistochemical stains for cytokeratins. The bar graph summarizes the relative number of bile ducts from WT (*n* = 9), YAP^KO^ (*n* = 8), TAZ^KO^ (*n* = 13), and DKO (*n* = 5) mice. ANOVA with Dunnett’s multiple comparisons test. ***p ≤ 0.001, **p ≤ 0.01. **G** Exemplary sirius red stains for the detection of extracellular collagen. The bar graph summarizes the normalized positive area for WT (*n* = 6), YAP^KO^ (*n* = 10), TAZ^KO^ (*n* = 7), and DKO (*n* = 7) mice. ANOVA with Dunnett’s multiple comparisons test. *ns* not significant. For **C**/**D**/**F**/**G**: Statistical comparisons not displayed do not reach the significance level (*p* > 0.05)

We designed mice with deletions of both genes alone or in combination in HCs and BECs (Alb-Cre x YAP^fl/fl^/TAZ^fl/fl^ resulting in YAP^KO^, TAZ^KO^, and YAP^KO^/TAZ^KO^ (termed as double knock-out: DKO)) (Suppl. Figure S1B-D) [25, 26]. As expected, the YAP target gene connective tissue growth factor (CTGF) was reduced in BECs (Suppl. Figure S1E). At 10 weeks, YAP-deficient animals showed liver-wide inflammation and parenchymal necrosis (YAP^KO^ and DKO) [21, 22, 27]. In contrast, genetically unmodified mice (WT) and TAZ^KO^ animals did not show this phenotype (Fig. 2A).

Interestingly, the number and size of these necrotic areas were significantly lower in livers isolated from DKO mice compared to YAP^KO^ animals (Fig. 2A/B, Suppl. Figure S1F). A moderately diminished phenotype in mice lacking both proteins compared to YAP^KO^ animals was also evident for the liver/body weight ratio as well as the liver damage markers alanine aminotransferase (ALT) and aspartate transaminase (AST) in serum from male and female mice (Fig. 2C/D, Suppl. Figure S1G/H). Indeed, the severity of necrosis in YAP^KO^ and DKO liver tissues strongly correlated with AST and ALT levels (Fig. 2E).

As hepatocellular damage is most likely caused by the loss of BECs after YAP/TAZ inactivation [1, 3], we hypothesized that the diminished phenotype was due to a less pronounced loss of BECs in DKO animals. Indeed, pan-cytokeratin (pan-CK) staining revealed that the number of bile ducts was higher in DKO mice compared to liver samples from YAP-deficient animals (Fig. 2F). No apparent bile duct morphology and density changes were detectable in TAZ-deficient animals. We assumed that the “milder” bile duct phenotype in DKO animals should also be reflected at the level of cholestasis-induced liver fibrosis. Indeed, the extent of fibrosis was moderately lower in DKO mice compared to YAP^KO^ animals (Fig. 2G).

One possible explanation for the partial rescue of the phenotype could be that YAP and TAZ compete for transcription factors as indicated in a recent study [28]. To confirm this mechanistically, we performed co-immunoprecipitation (coIP) experiments in a liver cancer cell line and detected YAP/TEAD and TAZ/TEAD complexes after gene-specific silencing of TAZ or YAP, respectively. Different coIP strategies revealed that inhibition of TAZ caused elevated interaction between YAP and TEAD family members. On the other hand, more TAZ/TEAD proteins were detectable after YAP inhibition (Suppl. Figure S2A–C). These results suggested that changes in the stoichiometry of the YAP/TAZ/TEAD complex support the formation of alternative transcriptional complexes. It is possible that TEAD in DKO mice regulates YAP/TAZ-independent target genes that counteract the YAP-deficiency phenotype.

Our findings show that TAZ cannot functionally compensate for the loss of YAP in BECs and HCs. Instead, TAZ deficiency counteracts the effects of YAP inactivation in HCs and BECs regarding the severity of liver necrosis, fibrosis, and cholestasis.

### YAP deficiency in HCs and BECs causes YAP activation in NPCs

**Fig. 3:**
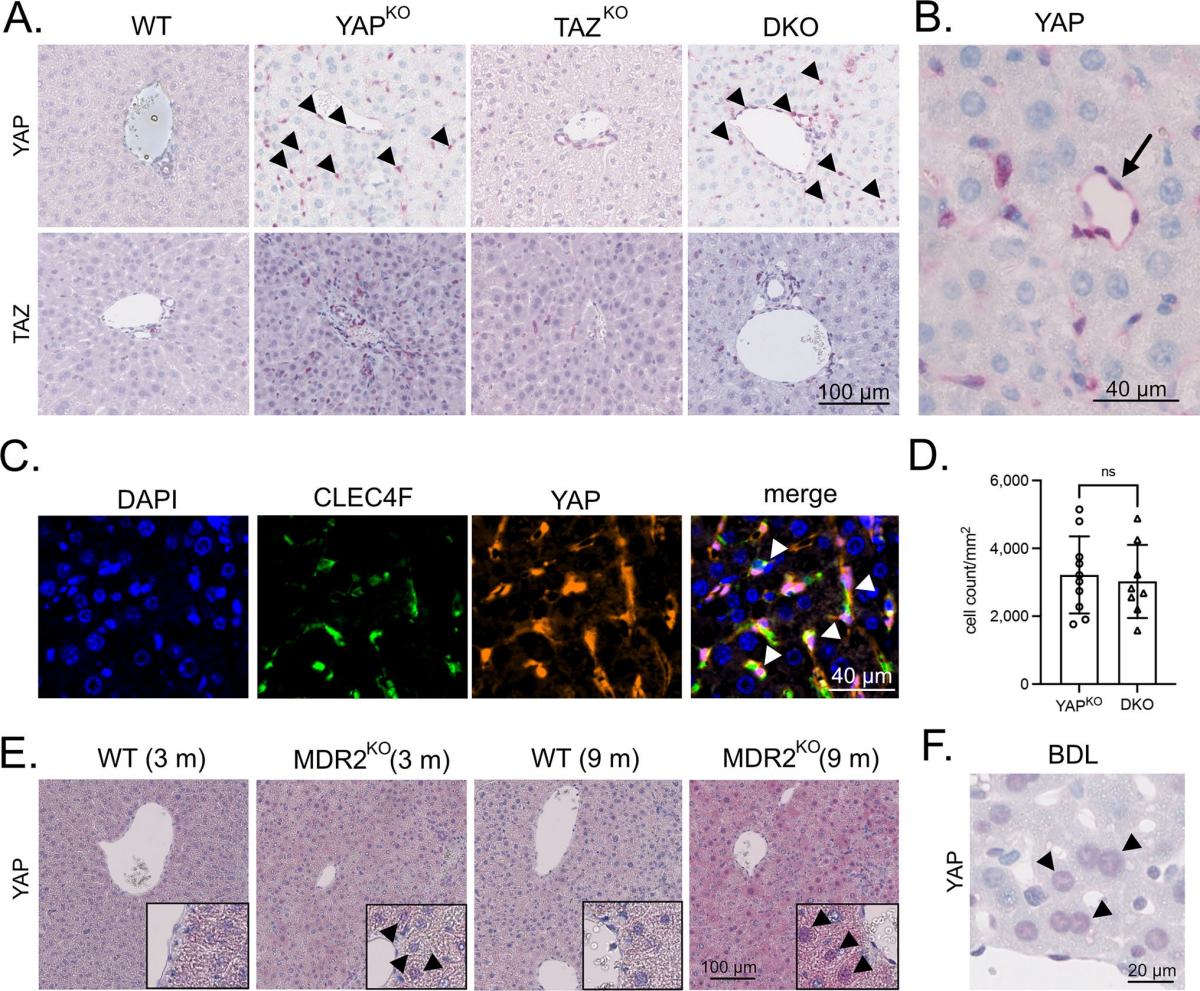
The absence of YAP in HCs and BECs induces YAP in NPCs.** A** Exemplary immunohistochemical stains for YAP and TAZ in WT, YAP^KO^, TAZ^KO^, and DKO liver tissues. Arrowheads point to YAP-positive sinusoidal cells of YAP^KO^ and DKO animals. No YAP positivity in NPCs was observed for WT or TAZ^KO^ samples. **B** High magnification picture of liver tissue derived from a YAP^KO^ mouse followed by YAP immunohistochemistry. While HCs were negative for YAP, a prominent expression of YAP was observed in ECs lining blood vessels (arrow). **C** Immunofluorescence stain of the KC marker CLEC4F (green) and YAP (red) in liver tissue from YAP^KO^ animals. Arrowheads: YAP positive KCs. **D** A machine learning algorithm was used to quantitatively compare YAP-positive NPCs in YAP^KO^ (*n* = 10) and DKO (*n* = 8) liver specimens. Statistical test: Mann–Whitney *U*. *ns* not significant. **E** Immunohistochemical staining of YAP in liver tissues from 3 and 9-month-old WT and MDR2^KO^ mice. Selected areas show HCs with nuclear YAP positivity (arrowheads). No consistent positivity for YAP was detected in NPCs. **F** YAP stain using liver tissues from mice after BDL. Arrowheads point to YAP-positive HC nuclei. No prominent YAP positivity for NPCs was detected

As YAP and TAZ showed different expression patterns in the sinusoidal cell compartment (Fig. 1), we analyzed YAP/TAZ expression in liver tissues derived from mouse lines with HC-/BEC-specific YAP-, TAZ-, and YAP/TAZ deficiency. While no noticeable YAP/TAZ positivity changes in TAZ^KO^ mice were detectable, a prominent induction of YAP in uniformly distributed sinusoidal cells was observed in YAP^KO^ and DKO animals (Fig. 3A). A mild induction of TAZ in sinusoidal cells from YAP^KO^ and DKO tissues was detectable, again pointing to distinct mechanisms that control YAP and TAZ in different liver cell types.

The YAP positivity in sinusoidal cells caused our particular interest as this observation indicated that YAP loss in HCs and BECs induced YAP in infiltrating immune cells or NPCs. Morphologically, YAP positivity, especially in ECs lining bigger vessels in the portal tract and the central vein, was evident for YAP^KO^ and DKO but not for WT and TAZ^KO^ mice (Fig. 3A/B, Suppl. Figure S3). Only a few cells in the liver sinusoids were YAP-positive in tissues from YAP^KO^ and DKO mice. We also tested if other NPCs showed elevated nuclear YAP expression after its inactivation in HCs and BECs. Indeed, co-staining with the KC marker CLEC4F confirmed YAP expression in this liver-resident myeloid cell population (Fig. 3C).

Next, we investigated if the YAP activation in NPCs is unspecifically caused by cholestasis due to the loss of BECs. In this case, less pronounced YAP positivity in NPCs from DKO tissues compared to YAP^KO^ livers would serve as a read-out for less severe liver damage observed earlier (Fig. 2). Surprisingly, the results revealed no significant difference in YAP positivity in sinusoidal cells, illustrating no association between the extent of cholestasis-induced liver damage and YAP induction in NPCs (Fig. 3D). We hypothesized that there may be other reasons besides cholestasis that can activate YAP in NPCs.

To substantiate these findings, we investigated liver tissues derived from multidrug resistance 2 (MDR2) knock-out animals (MDR2^KO^). These animals served as an independent model for cholestasis, characterized by leaky bile ducts, regurgitation of bile acids into the portal tract, and periductal fibrogenesis [29]. As described in the literature, cytoplasmic and nuclear YAP induction in HCs was observed in 3 and 9-month-old MDR2^KO^ mice, illustrating a permanent toxic stimulus associated with hepatocellular regeneration [21, 30, 31]. However, no consistent YAP positivity in the NPCs compartment was observed for both investigated time points (Fig. 3E). Equally, cholestasis-causing bile duct ligation (BDL)-induced YAP expression in HCs [32], while no significant YAP expression was detectable in NPCs (Fig. 3F).

In summary, YAP deficiency in liver HCs and BECs causes YAP expression in the NPC compartment. This activation relies on a cholestasis-independent mechanism.

### YAP is activated in NPC subclusters upon silencing in HCs and BECs

**Fig. 4:**
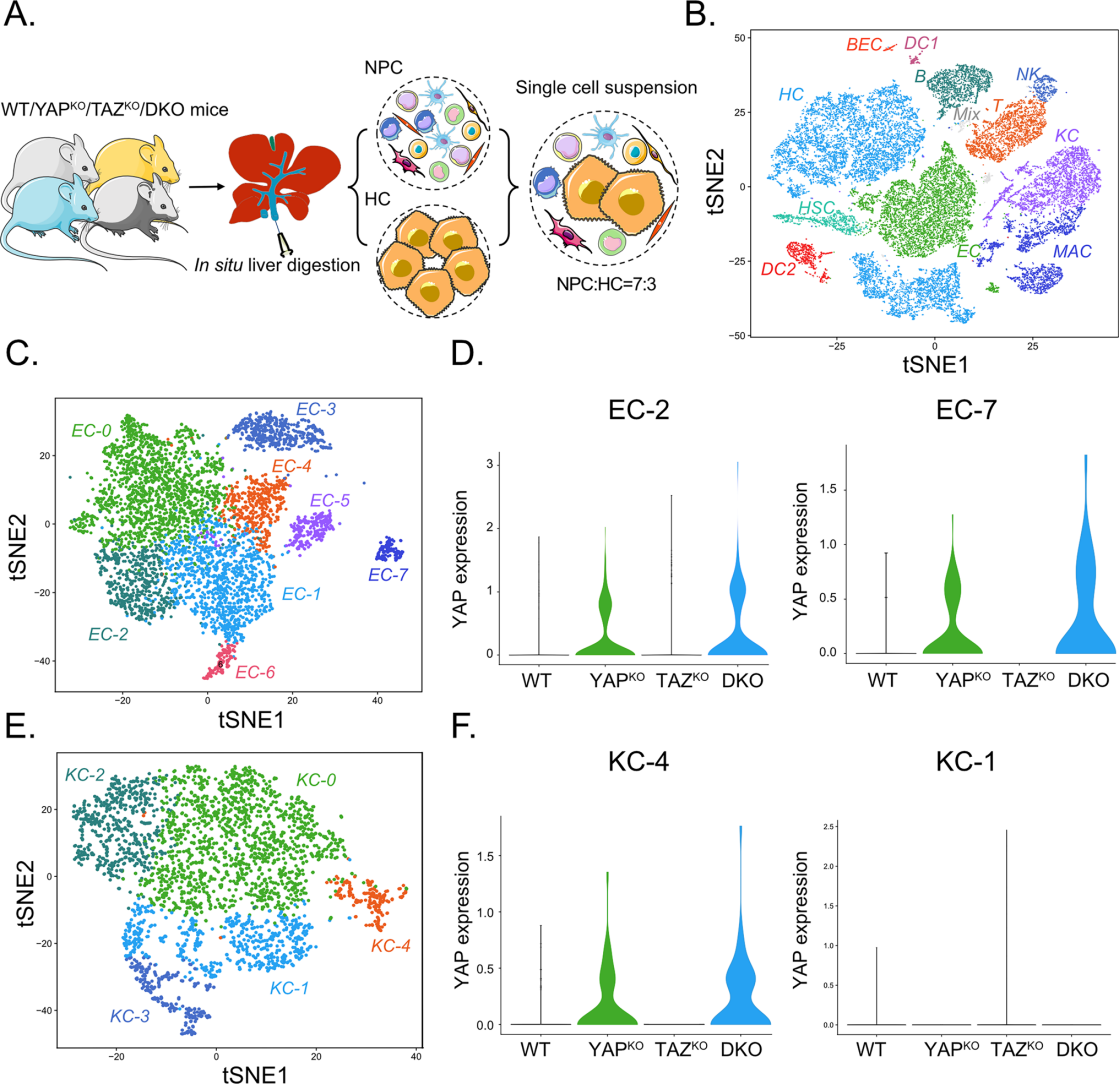
scRNA-seq reveals YAP induction in EC and KC subclusters.** A** Scheme illustrating the experimental setup. Livers from WT, YAP^KO^, TAZ^KO^, and DKO mice were digested in situ to achieve pure and viable murine HCs (20–30 µm), smaller NPCs, and infiltrating immune cells. Cell fractions were mixed at a ratio of 7:3 and applied to the 10x  scRNA-seq platform. Figure 4A was modified using Servier Medical Art (http://smart.servier.com). **B** T-distributed stochastic neighbor embedding (t-SNE) plot showing annotated and color-coded cell types derived from WT, YAP^KO^, TAZ^KO^, and DKO livers. *HC* hepatocytes, *HSC* hepatic stellate cells, *NK* natural killer cells, *B* B cells, *MAC* macrophages, *DC1* dendritic cells cluster 1, *BEC* biliary epithelial cells, *EC* endothelial cells, *KC* Kupffer cells, *DC2* dendritic cells cluster 2, *Mix* cells not expressing distinct marker genes. **C** t-SNE plot illustrating eight subtypes of ECs. The different EC subclusters are characterized by specific marker genes and cellular processes such as EC-0 (CDH13, WNT2, KIT, PLPP1 representing pericentral ECs) and EC-1/EC-6 (EFNB1, LTBP4, LY6A, NTN4, representing periportal ECs). **D** Violin plot showing YAP induction in the subclusters EC-2 and EC-7 from YAP^KO^ and DKO animals but not WT and TAZ^KO^ mice. Other EC subclusters did not show a comparable YAP expression pattern. **E** t-SNE plot illustrating five subtypes of KCs. The different KC subclusters are characterized by specific marker genes and cellular processes, such as KC-0 (M1 polarization-like phenotype) and KC-1 (lipid metabolism). **F** Violin plot for subcluster KC-4 demonstrating the induction of YAP in liver tissues from YAP^KO^ and DKO mice. Other KC subclusters did not show a comparable YAP pattern (exemplified for KC-1)

To further investigate YAP induction in ECs and KCs, we performed scRNA-seq analysis using liver tissues from WT, YAP^KO^, TAZ^KO^, and DKO mice. For this, we established a two-step in situ digestion protocol that allowed the isolation of viable NPC and HC cell suspensions in a defined ratio (Fig. 4A, Suppl. Figure S4). The NPC/HC cell mixture was subjected to scRNA-seq analysis followed by unbiased clustering of 26,746 cells. Eleven distinct liver cell types were identified, including HCs, BECs, ECs, and KCs, and infiltrating immune cells such as T cells or macrophages (Fig. 4B, Suppl. Figure S5). BECs were not efficiently isolated using this protocol, which explains their low numbers for WT and TAZ^KO^ mice.

It is well accepted that liver-resident ECs and KCs consist of subtypes with distinct molecular features [33, 34]. Indeed, unsupervised clustering of ECs from all four mouse lines revealed the existence of eight EC subclusters (EC-0 to EC-7) (Fig. 4C, Suppl. Figure S6). For example, cluster EC-0 was characterized by the expression of cadherin-13 (CDH13) and Wnt family member 2 (WNT2) and showed features of pericentral ECs [35]. In contrast, cells of EC-1/EC-6 were characterized by high expression of ephrin B1 (EFNB1), late transforming growth factor beta-binding protein 4 (LTBP4), basal cell adhesion molecule (BCAM), thrombomodulin (THBD), as well as von Willebrand Factor (VWF) and showed features of periportal, continuous ECs [34, 35]. EC-4 cells represented LSECs because stabilin1 (STAB1), stabilin2 (STAB2), EH domain containing 3 (EHD3), and FC gamma receptor IIb (FCGR2B) were expressed.

Importantly, we observed an induction of YAP transcripts exclusively in subclusters EC-2 and EC-7 derived from YAP^KO^ and DKO mice (Fig. 4D). Such a pattern was not detectable in other EC subclusters derived from YAP^KO^ and DKO animals, such as EC-0, EC-1, EC-3, EC-4, EC-5, and EC-6 (Suppl Figure S7A). YAP-positive EC-2 cells were characterized by high expression of several interferon-inducible genes such as interferon-induced protein with tetratricopeptide peptides 1 (IFIT1), IFIT2, IFIT3, and interferon-induced protein 44 (IFI44), (20/114 significantly regulated genes in EC-2 were IFN-regulated; Suppl. Figure S6). Indeed, C-X-C motif chemokine ligand 10 (CXCL10), a known interferon target gene [36], was equally expressed in this subcluster EC-2. The YAP positive subcluster EC-7 was characterized by the expression of several S100 protein family members (e.g., S100A9, S100A8) and C-X-C motif chemokine ligand 2 (CXCL2) expression, which are known pro-proliferative factors for ECs [37, 38]. Thus, cluster EC-7 showed features of mitotically active cells. We observed no significant enrichment of known YAP target genes in any of the EC subclusters, which was further validated in vitro using different EC cell lines (data not shown).

For KCs, five subclusters were detectable (KC-0 to KC-4) (Fig. 4E, Suppl. Figure S8). For example, subcluster KC-3 was characterized by high expression of the proliferation makers KI67 (MKI67) and microtubule-destabilizing factor stathmin (STMN1). They could represent a mitotically active source of liver-resident KCs. As shown for the EC compartment, YAP induction was not detectable in all KCs but exclusively in the subcluster KC-4 derived from YAP^KO^ and DKO liver tissues (Fig. 4F, Suppl. Figure S7B). The YAP positive cluster KC-4 was characterized by high expression of several paracrine-acting factors, including secreted protein acidic cysteine-rich (SPARC) and bone morphogenetic protein 2 (BMP2), indicating that this group of KCs might be of particular importance in heterologous cell communication [39].

Interestingly, no prominent induction of YAP in HSC subtypes was detectable (Suppl. Figure S9). These results suggested that the described mechanism for YAP expression in HSC activation differs from processes observed in our study for ECs and KCs [40]. However, we identified HSC subpopulations, which have been previously described in hepatocarcinogenesis [41].

In summary, the scRNA-seq data confirm the induction of YAP expression in NPCs such as ECs and KCs after genetic inactivation of YAP in HCs and BECs. YAP is not uniformly expressed in NPCs but in distinct EC and KC subtypes with specific molecular and biological features.

### YAP activation in NPCs is associated with a pro-inflammatory secretome

As YAP was activated in a subgroup of NPCs, we hypothesized that this induction contributed to the inflammatory response observed in YAP^KO^ and DKO animals. To investigate whether YAP-positive EC subclusters express cytokines and chemokines that could affect inflammation, we performed Gene Ontology pathway analysis using scRNA-seq data from subclusters EC-2 and EC-7. Significant enrichment of genes involved in regulating the immune cells was detected, such as “regulation of inflammatory response” (GO:0050727) (not shown). In detail, several chemoattractants such as CXCL10 (EC-2 and EC-7), IL1B (EC-7), and CXCL2 (EC-7) were induced (Suppl. Figure S10A). In addition, factors involved in pro-inflammatory cellular responses were equally enriched, such as S100 family members (EC-7, Suppl. Figure S10B).

**Fig. 5:**
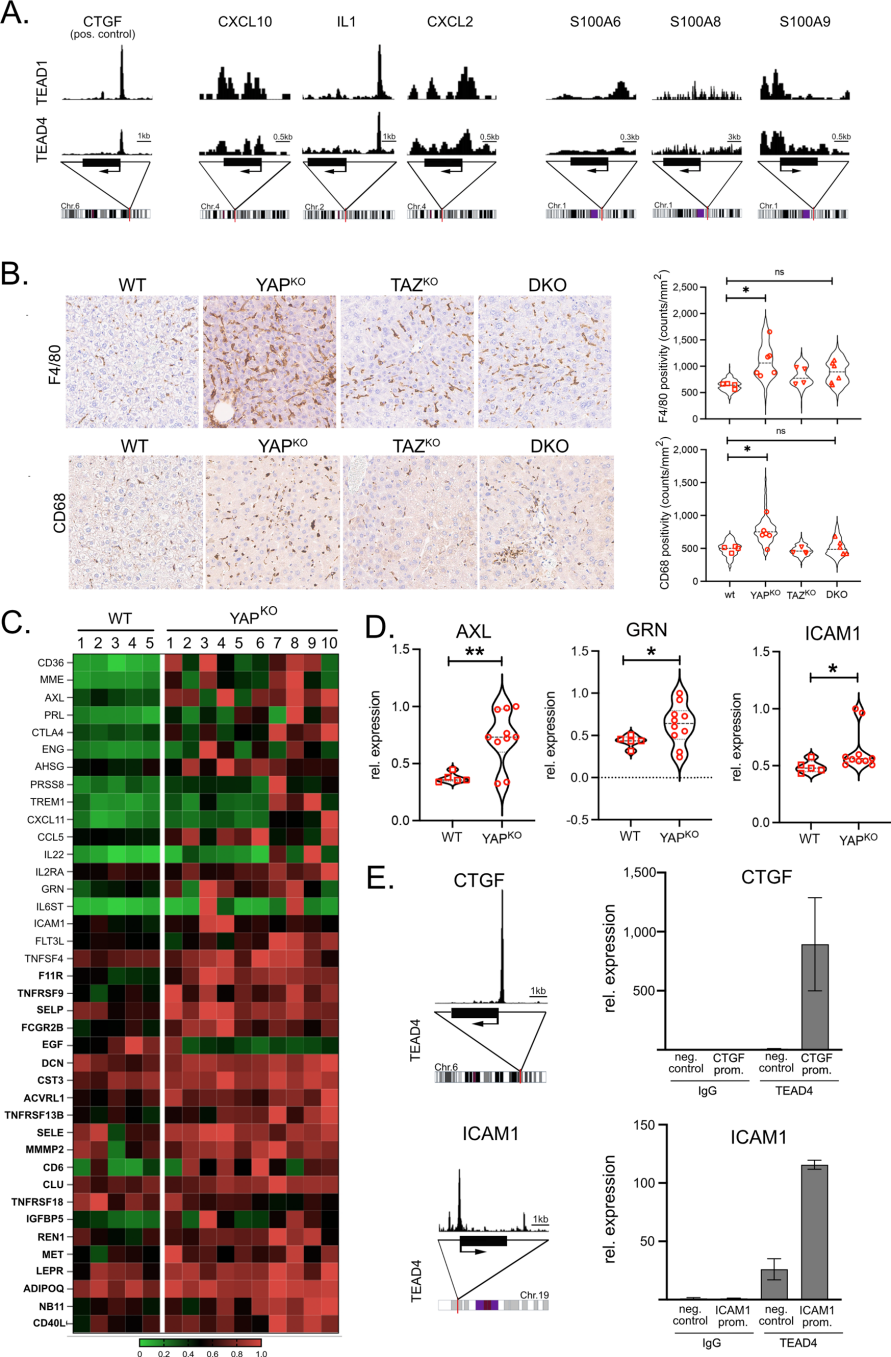
Expression of pro-inflammatory chemokines in YAP-deficient mice.** A** Scheme depicting TEAD1 and TEAD4 ChIP-Seq profiles in the promoter regions of human CTGF (positive control), CXCL10, IL1, CXCL2, S100A6, S100A8, and S100A9. **B** Immunohistochemical staining of the KC/macrophage markers F4/80 and CD68. Violin plots illustrate respective quantification. WT (*n* = 4), YAP^KO^ (*n* = 6), TAZ^KO^ (*n* = 4), and DKO (*n* = 6) animals were analyzed using a machine learning algorithm. In total, 1.408 (F4/80) and 1.220 (CD68) tiles were quantitatively investigated. Statistical test: ANOVA with Dunnett’s multiple comparisons test. *p ≤ 0.05, ns: not significant. Statistical comparisons not displayed do not reach the significance level (*p* > 0.05). **C** Heatmap summarizing secreted factors (cytokines, chemokines, and growth factors) in WT and YAP^KO^ mice serum. Factors were measured using a mouse cytokine array (WT: *n* = 5, YAP^KO^: *n* = 10). Samples were first tested for equal or unequal variances (F-test). For samples with F < 0.05 (different variances; *n* = 18) or F > 0.05 (identical variances, *n* = 21 in bold), the respective statistical tests were performed. Out of the 200 factors that were measured, 39 displayed significant differences (*p* ≤ 0.05). **D** Exemplary violin plots of AXL, GRN, and ICAM1 in WT and YAP^KO^ mice serum samples are shown. *p ≤ 0.05, **p ≤ 0.01. **E** Analysis of TEAD4 ChIP-seq data derived from liver cells. The YAP target gene CTGF was used as a positive control. Results showed TEAD binding sites in promoter regions of the ICAM1 gene. A ChIP experiment for detecting binding sites in the ICAM1 promoter was performed with TEAD4 in HLF cells. CTGF served as a positive control. For ICAM1 and CTGF, upstream promoter regions served as negative controls (neg. control). IgG was employed as antibody control

As the expression of genes in subclusters EC-2 and EC-7 does not prove YAP/TEAD dependency, we analyzed genomic ChIP-seq data for the presence of binding sites for TEAD family members in a panel of identified pro-inflammatory factors. For CXCL10, CXCL2, S100A6, and S100A9, enrichment of TEAD1/4 binding close to the respective gene promoters was detectable (Fig. 5A). For S100A8, no apparent TEAD1/4 binding was observed.

As the YAP-associated expression of pleiotropic chemokines by ECs suggested effects on leucocytes in YAP^KO^ and DKO mice, we quantitatively characterized the abundance of several immune cell types by immunohistochemistry (Fig. 5B, Suppl. Figures S11/S12). The results illustrated that the number of some cell types, such as CD3- and CD4-positive T cells, slightly increased in YAP^KO^ and DKO animals; however, this response did not reach the level of significance compared to WT mice (Suppl. Figure S12). In contrast, significantly more KCs/macrophages (markers: F4/80, CD68; Fig. 5B), granulocytes (marker: myeloperoxidase; MPO), B cells (marker: B220), and CD8 cells were detected in YAP^KO^ animals (Suppl. Figure S12). This attraction of immune cells was less pronounced in tissues derived from TAZ^KO^ mice, illustrating that TAZ deficiency in HCs and BECs led to a mild immune response. Interestingly, the number of some immune cell types, such as macrophages and B cells was diminished in DKO animals compared to YAP^KO^ animals, which was in line with the mitigated DKO phenotype observed before.

To substantiate the association of YAP-positive NPCs and induction of a secretory phenotype at the protein level, we compared the cytokine/chemokine abundance in the serum of WT and YAP^KO^ mice using a cytokine antibody array. In total, 39 significantly regulated cytokines/chemokine/growth factors were identified, including known YAP target genes (e.g., AXL, [42]) as well as proteins known to regulate fibrosis (e.g., progranulin (GRN, [43])), or hepatic inflammation (e.g., intercellular adhesion molecule-1 (ICAM1), which exists as shedded and soluble protein [44, 45])), (Fig. 5C/D). To clarify if the YAP/TEAD complex could transcriptionally control these secreted factors, we searched for binding sites of TEAD family members using ChIP-seq data [46, 47]. Indeed, for about 53% of the secreted factors, TEAD binding sites in the respective gene promoters were detectable such as C-X-C motif chemokine ligand 11 (CXCL11) and fms-related tyrosine kinase ligand (FLT3L), and ICAM1 (GEO accession: GSE170161). Exemplarily, the interaction of TEAD4 with the DNA binding site was confirmed by chromatin immunoprecipitation (ChIP) for ICAM1 (Fig. 5E). As illustrated for CXCL11 and FLT3L in vitro, these factors could contribute to the induction of YAP in myeloid and endothelial cells (Suppl. Figure S13A/B) and the migratory capacity of an immortalized KC line (Suppl. Figure S13C).

These findings support the hypothesis that YAP activation in NPCs contributes to transcriptionally regulated changes in the secretome that impact the pro-inflammatory response.

## Discussion

The Hippo pathway effectors YAP and TAZ play important roles in maintaining tissue homeostasis during regeneration and are also involved in the development of various types of tumors. Although both transcriptional regulators bind TEAD proteins and similar sets of gene enhancers [7], accumulating evidence illustrates non-redundant YAP and TAZ properties under physiological and disease conditions [6, 8, 9, 48–50]. Because the first anti-cancer drugs interfering with YAP/TAZ/TEAD activity have entered clinical trials, we aimed to investigate the steady-state and dynamic expression of YAP and TAZ after their inactivation in hepatocytes and cholangiocytes, which represent the central functional units of the liver. We systematically compared the functional impact of YAP and/or TAZ depletion and investigated how they control paracellular communication with a focus on NPCs.

Our results show that YAP and TAZ are not equally expressed in parenchymal and non-parenchymal cells in healthy liver tissue. According to our and previous data, YAP is detectable in BECs, weakly expressed in HCs, and absent in NPCs [51]. The loss of YAP in epithelial cells causes a phenotype characterized by severe liver damage associated with the loss of BECs, cholestasis, and hepatocellular necrosis [21, 22, 27]. In contrast, TAZ protein is not expressed in the hepatic epithelial cell compartment but in small sinusoidal cells. However, low TAZ amounts could contribute to BEC and HC biology and synergize after simultaneous YAP deletion. Surprisingly, many (but not all) YAP depletion-induced phenotypes were partially antagonized by simultaneous inactivation of TAZ (YAP^KO^ vs. DKO, e.g., parenchymal necrosis and immune cell infiltration). To our knowledge, this is the first time that counteracting properties of TAZ depletion have been demonstrated. Notably, our molecular analyses illustrate that inhibition of YAP or TAZ changes the stochiometry of TAZ/TEAD or YAP/TEAD complexes, respectively. This finding is supported by a previous publication showing elevated TAZ/TEAD binding in YAP-deficient cells [28]. It is tempting to hypothesize that unbound TEAD molecules in cells with combined YAP/TAZ inhibition facilitate YAP/TAZ-independent (cytoprotective) effects, which counteract liver damage and inflammation observed in YAP^KO^ mice.

Interestingly, previous data illustrated that YAP/TAZ double knock-out mice showed stronger bile duct loss than YAP^KO^, which pointed to additive or synergistic effects of YAP and TAZ [3]. However, this phenotype in YAP^KO^ or DKO mice was not quantitatively described. For this reason, the protective effects we observed might be missed in former studies since non-quantitative approaches might be insufficient to detect subtle changes. For this reason, we applied semi-automatic quantitative methods on stained tissues, demonstrating the antagonizing impact of TAZ deficiency in DKO mice for parenchymal necrosis, bile duct loss, fibrosis, and infiltration of some immune cells. Although our current study did not further decipher the underlying mechanism, different molecular or cellular scenarios explaining the antagonizing effects of TAZ depletion are possible. First, the inactivation of low but transcriptionally active TAZ amounts in BECs or HCs counteracts YAP deletion via the regulation of TAZ-dependent target genes. Second, although not causing a noticeable loss of BECs, TAZ depletion in HCs and BECs leads to a mild inflammatory response (e.g., elevated myeloid and T cells). This immune response may differ from one caused by YAP depletion and may counteract the phenotype observed in YAP^KO^ mice. The explanation is supported by our observation that under regenerative conditions without inflammation (PHx), this protective effect of TAZ depletion is not detectable (data not shown). Third, stoichiometry changes after YAP/TAZ inactivation partners could affect the transcriptional activity of TEADs (see discussion above). For example, TEADs that are not forming complexes with YAP/TAZ could interact with TCF7L2, also known as TCF4, to regulate alternative gene expression [52].

Another important observation of our study was the expression of YAP in different liver-resident cells upon its genetic inactivation in HCs and BECs. Indeed, the loss of BECs is characterized by cholestasis and bile acids that activate YAP expression in HCs [31]. As we could rule out that cholestasis causes YAP induction in NPCs, specific paracrine communication networks or physical cell–cell interaction likely contribute to this phenomenon. Indeed, it is known that the Hippo pathway actively controls the cellular secretome and cell–cell contact proteins [6, 53]. Our preliminary data suggest that CXCL11 and FLT3L, which are elevated in YAP^KO^ mice serum, may contribute to YAP induction in myeloid and endothelial cells as well as KC migration. This observation is important for designing future YAP/TEAD-directed therapies for two reasons. First, systemic therapies would block YAP/TEAD activity in tumor cells with aberrant YAP expression and other cell types, such as hepatic BECs (which could lead to cholestasis) and NPCs (with effects on heterologous cell communication). As suggested by our scRNA-seq data, this could affect EC- and KC with YAP-dependent molecular features. Second, perturbation of YAP in different cell compartments may not necessarily cause anti-tumorigenic effects but can also support carcinogenesis, as illustrated for breast cancer and hepatocarcinogenesis [54–57]. For instance, the relative activity of YAP in cancer cells and non-tumorous hepatocytes defines tumorigenicity, suggesting that the biological output of YAP is regulated on the level of individual cell types [56]. Future studies must investigate which YAP effects dominate in distinct cell types before specific drugs are applied to cancer patients.

YAP neo-expression after its deletion in HCs/BECs was detectable in different sinusoidal cells. Histomorphological, biochemical, and molecular analyses illustrated that ECs and KCs but not HSCs belong to this group of YAP-positive cells. However, the dissemination of YAP-positive cells in YAP^KO^ and DKO animals indicated that not all ECs were characterized by YAP expression, e.g., LSECs lining the sinusoids mainly were negative, while ECs lining bigger vessels stained positive for YAP. Our scRNA-seq results supported this finding, demonstrating that EC/KC subtypes with specific molecular features were characterized by YAP expression. For example, YAP positivity in ECs/KCs was associated with the expression of paracrine-acting factors and chemoattractants, confirming YAP’s pro-inflammatory role [58, 59]. Subcluster EC-2 is characterized by the expression of interferon-inducible genes such as IFI or IFIT family members and CXCL10, indicating that this EC cluster responds to systemic interferon [36]. Thus, this cluster may contribute to CXCL10-mediated liver fibrosis and activation of immune cells [60]. Alternatively, these ECs might be involved in intracellular crawling, which describes lymphocyte migration in liver ECs upon interferon stimulation [61]. Interestingly, many well-described YAP target genes, such as CTGF and CYR61, were not statistically enriched in any of the EC subclusters, indicating that YAP controls an alternative gene signature in this cell type.

This study reveals a comprehensive overview of YAP and TAZ biology upon their inactivation in HCs and BECs. First, TAZ cannot compensate for YAP deficiency and its deletion causes mild effects on inflammation. In contrast, TAZ inactivation partly counteracts effects caused by YAP inhibition. Second, YAP is induced in ECs and KCs with specific molecular features and may contribute to an inflammatory process. These observations are important for future systemic anti-cancer therapies as drugs will also target the activity of YAP and TAZ in non-malignant cells.

## Materials and methods

### Mouse models and generation of knock-out mice

Animal work on mouse models was authorized by the Regional Councils of Baden-Württemberg (ref. numbers: G-307/14, G-53/20). All experiments were performed in accordance with the regional institutional regulations in the animal facilities of the University of Heidelberg, the German Cancer Research Center (DKFZ), and the Hannover Medical School (MHH) under pathogen-free conditions. The mouse colonies were housed under a 12 h light/dark cycle with free access to water and food. Local animal welfare regulations defined criteria for the exclusion of animals and/or the termination of experiments.

The generation of mice carrying the conditional *Yap*-loxP and *Taz*-loxP alleles has been described previously [25]. These mice were crossed with animals expressing albumin-regulated Cre recombinase (Alb-Cre) to generate mice with HC- and BEC-specific knock-out of YAP and/or TAZ [26]. For genotyping of wildtype (WT), YAP^KO^, TAZ^KO^, and double knock-out (DKO) mice, DNA was extracted from ears or liver samples followed by PCR analysis (primers listed in supplementary Table S1) [25]. The PCR product size for the *Yap* gene in WT, *loxP*-floxed, and KO alleles were 457 bp, 600 bp, and 338 bp, respectively. For the *Taz* gene, 496 bp, 655 bp, and 704 bp products were detected. PCR was also used to test for Cre recombinase positivity (700 bp). The following PCR program was used: 1 × 94 °C (5 min); 35 × 94 °C (30 s), 65 °C (45 s), 72 °C (90 s); 1 × 72 °C (10 min). For the Cre recombinase product detection, the annealing temperature was reduced to 58 °C. PCR products were loaded and separated by electrophoresis using a 2% agarose gel. DNA fragments were detected using a LI-COR D-Digit gel scanner (LI-COR Biosciences, Bad Homburg, Germany).

Generation of multidrug resistance 2 (MDR2)-negative mice by homozygous deletion of the *Mdr2* gene was previously described [62, 63]. Mice were sacrificed at the age of 3 and 9 months. All animal experiments were approved by the German Regional Council of Baden-Württemberg (Regierungspräsidium Karlsruhe, ref. number: G-21/17).

Bile duct ligation (BDL) was performed as previously described [64]. The respective authority approved animal experiments for animal experiments (district government of Lower Saxony, Germany, ref. number: 33.9-42,502-04-08/1580).

### Partial hepatectomy, CCl4-induced liver damage, and tissue sampling

*Partial hepatectomy* (70% PHx) was performed using 10-week-old WT male animals. In brief, mice were anesthetized and 2/3 of the liver was removed after laparotomy. For this, the left lateral lobe and the median lobe were surgically removed after ligation. Afterward, the peritoneum and skin were closed. Carprosol (5 mg/kg body weight) was given subcutaneously after surgery, followed by further treatments every 8 h for 2 days. Tissues were collected 2, 4, and 9 days after PHx.

To induce fibrosis, 10-week-old WT mice received intraperitoneal carbon tetrachloride (CCl_4_) injections (667 μl/kg body weight, 2x/week for 6 weeks). CCl_4_ was diluted in mineral oil with a ratio of 1:5. Samples were taken 4 weeks after the last injection (age of mice: 20 weeks). Mice from the control group were injected with mineral oil.

For the isolation of serum, the following protocol was used. After retro-orbital sampling with anesthesia (1.5% isoflurane with 500 ml/min oxygen), blood was centrifuged at 10,000 g for 5 min, and serum was collected for the measurement of the liver damage markers alanine transaminase (ALT) and aspartate transaminase (AST).

For tissue sampling, the abdomen was carefully opened, and the liver was removed after cutting the liver ligament. After washing with phosphate-buffered saline (PBS), liver tissues were immediately fixed in 4% formalin for paraffin embedding. Additional liver pieces were embedded in Tissue-Tek OCT (Science Services, Munich, Germany) and transferred into liquid nitrogen. The remaining liver tissues were frozen immediately in liquid nitrogen for mRNA and protein isolation. All samples were stored at -80˚C.

### Real-time PCR analysis

Real-time PCR was performed using the ABsolute qPCR SYBR Green Mixes according to the manufacturer’s protocol and the StepOnePlus™ Real-Time PCR system (Thermo Fisher Scientific). Glycerinaldehyd-3-phosphat-dehydrogenase (GAPDH), peptidylprolyl isomerase A (PPIA), tubulin, and TATA-box binding protein (TBP) were used for the normalization of data derived from mouse tissues. The standard curve method was used for quantification analysis (primers listed in supplementary Table S1).

### Cell culture and siRNA transfection

The hepatocyte-derived cell line HLF was obtained from the Japanese Collection of Research Bioresources (JCRB, Osaka, Japan). SVEC4-10 and RAW267 cells were obtained from ATCC (LGC Standards). Cell lines were cultured in DMEM medium (Sigma Aldrich, Steinheim, Germany) supplemented with 10% fetal bovine serum (FCS) and 1% penicillin/streptomycin. Cells were maintained at 37 °C and 5% CO_2_ in a humidified atmosphere and routinely tested for mycoplasma contamination. Authentication was performed by short tandem repeat analysis (DSMZ, Braunschweig, Germany).

For transient transfection of gene-specific small interfering RNA (siRNA, Microsynth, Göttingen, Germany), Oligofectamine was used according to the manufacturer’s protocol (Thermo Fisher Scientific, Darmstadt, Germany). siRNAs were used at a final concentration of 20 nM. For transfection, three 10 cm dishes with 7 × 10^5^ cells were prepared one day before transfection. Equimolar concentrations of no template control (NTC) and YAP and TAZ-specific siRNAs were used. The cell culture medium was replaced after 24 h. siRNAs used in this study were: no-template control (NTC): 5'-UGG-UUU-ACA-UGU-CGA-CUA-A-dTdT-3'; siYAP: 5'-GGA-GGA-AGC-UAG-AUA-AAG-AUU-dTdT-3'; siTAZ: 5'-AGG-UAC-UUC-CUC-AAU-CAC-A-dTdT-3'.

For stimulation experiments, SVEC4-10 cells were starved overnight and stimulated with murine CXCL11 (100 ng/ml, R&D Systems, Minneapolis, MN, USA) and FLT3L (100 ng/ml, R&D Systems) for 24 h. Isolated proteins were subjected to Western immunoblotting.

RAW264 cells were starved for 12 h and treated with CXCL11 and FTL13 (100 ng/ml). Following a 24 h incubation, the medium was removed, and the cells were washed three times with PBS. The cells were then fixed with paraformaldehyde and treated with 0.1% Triton-X for 20 min. After three washes with PBS, the cells were blocked with goat serum for 1 h at RT. Cells were incubated with the YAP antibody for 16 h at 4 °C. After washing with PBS, the second antibody was added for 1 h. The nuclei were stained with DAPI.

Immortalized KC cells (ImKCs) were obtained from MilliporeSigma/Merck KGaA, Darmstadt, Germany) and cultured in RPMI medium supplemented with 10% fetal bovine serum and 1% penicillin/streptomycin [65].

### Lateral migration experiment

ImKCs were seeded in ibidi inserts (ibidi GmbH, Gräfelfing, Germany) creating a 500 µm gap. In the evening, FCS was reduced by changing the culture medium to 1% FCS with antibiotics. The next day, mitomycin was added to block cell proliferation (2.5 µg/ml medium). After incubation for three hours, the ibidi inserts were removed and new medium containing 1% FCS and cytokines was added (100 ng/ml for CXCL11 and FLT3L, respectively). Digital pictures of gaps were taken immediately after removing the inserts (0 h) and after 48 h.

### Western immunoblotting and co-immunoprecipitation (coIP)

Extraction of total protein extracts and analysis by Western immunoblotting and coIP was recently described in detail [66]. Proteins for coIP experiments were isolated 48 h after siRNA transfection. Antibodies used in this study are listed in Suppl. Table S2. Signal detection for coIP experiments was done using the Quick Western Kit (LI-COR Biosciences, Bad Homburg, Germany).

### Chromatin immunoprecipitation (ChIP)

HLF cells were seeded onto 15 cm dishes and incubated until reaching about 80% confluency. Cells were fixed with 1% formaldehyde in PBS for 15 min and quenched with 2.5 M glycine for 5 min. Subsequently, cells were harvested in RIPA buffer supplemented with 1 × Protease Inhibitor Mix G and sonicated to generate DNA fragments of less than 500 bp. After preclearing, samples were mixed with 2 µg of specific antibody or IgG as control and Dynabeads, followed by incubation at 4 °C overnight. After several washing steps (4 × RIPA, 4 × IP wash buffer, 2 × TE), the protein-DNA complexes were eluted from the Dynabeads. Cross-linking was reversed by adding 4 M NaCl and incubation at 65 °C for 5 h. DNA was purified using the NucleoSpin® Gel and PCR Clean-up Kit according to the manufacturer’s instructions. Finally, precipitated DNA was quantified with qPCR using a serial dilution of genomic DNA to calculate a reference standard curve. ChIP primers were designed based on the TEAD4 binding sites identified by ChIP-Seq data analysis and the prediction of TEAD4 binding sites using the JASPAR database [67]. Commercially available primers covering the human CTGF promoter and primers covering the CTGF upstream region without transcription factor binding site were employed as positive and negative controls, respectively (SimpleChIP®, Cell Signaling).

### Tissue staining protocol

After overnight fixation with 10% buffered formalin, liver specimens were dehydrated, embedded in paraffin, and cut into 2–3 μm sections. All tissue sections were stained with H&E and counterstained with hematoxylin according to a standard protocol.

For immunohistochemistry, sections were deparaffinized and rehydrated: 3 × xylene (5 min), 2 × 100% ethanol (2 min), 2 × 95% ethanol (2 min), 2 × 70% ethanol (2 min), rinsing with aqua dest. Antigen retrieval was done using a pressure cooker (YAP, TAZ) or steamer (pan-CK, CTGF) in citrate buffer. For staining YAP and TAZ, slides were incubated with primary antibodies at 4 °C overnight (antibodies are listed in Suppl. Table S2). Slides were washed in TBST buffer twice for 5 min, and tissue sections were incubated with Enzo anti-Rabbit Polymer-AP for 30 min (Enzo Life Sciences GmbH, Lörrach, Germany). Signal detection was done with liquid Permanent Red (Agilent/Dako, Santa Clara, CA, USA). For pan-CK detection, horseradish peroxidase (HRP) was utilized. Slides were blocked with H_2_O_2_ for 7 min, followed by incubation with the first antibody at room temperature for 1 h. The Polyview Plus HRP system was used for signal detection (Enzo Life Sciences GmbH). For the immune marker staining, the fully automated BOND system was used (Leica Biosystems, Wetzlar, Germany).

Sirius red staining was performed for visualization of extracellular connective tissue material. For this, dewaxed and rehydrated tissue slides were immersed in ready-to-use sirius red solution for 1 h (Morphisto GmbH, Frankfurt am Main, Germany). Slides were washed with hydrochloric acid twice for 1 min. All slides were mounted with coverslips.

### Machine-learning algorithm and image analysis

Stained tissue slides were used for image analysis and quantification. Sections were digitalized using slide scanners (Aperio AT2, Leica Mikrosysteme Vertrieb GmbH, Wetzlar, Germany or ZEISS Axio Scan Z1, Carl Zeiss Microscopy GmbH, Oberkochen, Germany) at 40 × magnification. The whole slide images were divided into tiles (square images of 1 mm^2^ size) using OpenSlide in Automated Slide Analysis Platform (ASAP) software (https://computationalpathologygroup.github.io/ASAP/). All tiles except those with evident staining and/or scanning artifacts were used for image processing and quantification. Furthermore, tiles with half or less of viable tissue area were excluded.

A random forest machine-learning algorithm was trained to recognize stained immune cells or cytoplasmic stains (e.g., sirius red stains) on the tissue samples using the Ilastik software (v1.3.3) [68]. The random forest classifier was applied to all tiles to generate probability maps. The resulting probability maps were thresholded and quantified with ImageJ scripts (v1.53q) [69].

Immunofluorescent stainings of YAP and DAPI were documented with 40X magnification. For each treatment group, the fluorescence intensity of four representative pictures was quantified using ImageJ. To quantify YAP intensity, all pictures were adjusted with the same threshold. For cell counting, the DAPI channel was adjusted with the same threshold and then converted to binary using the “make binary” function. After applying the “fill holes” and “watershed” processes, the “analyze particles” function was used to quantify the cells. The cell count normalized the YAP intensities, and statistical analysis between different groups was performed using One-way ANOVA.

### Mouse cytokine antibody array

Two hundred cytokines and growth factors in the serum of mice were measured using the G-series mouse cytokine antibody array 4000 (Raybiotech, Norcross, GA, USA). Five hundred microliter of serum from 5 WT and 10 YAP^KO^ mice were quantitatively measured (tebu-bio, Perray en Yvelines, France). Before calculating the p-value, results for each cytokine in WT and YAP^KO^ were tested for equal variances (F-test).

### Measurement of blood serum markers

For the analysis of liver damage markers, 10 µl of murine blood serum was applied to Fuji DRI-Chem slides (GPT/ALT and GOT/AST) and measured automatically using the DRI-Chem NX500 (Fuji GmbH, Tokyo, Japan).

### Cell isolation for single-cell analysis

Mice were anesthetized with ketamine/xylazine (100 mg/kg and 12 mg/kg, respectively). After skin disinfection, the abdomen was opened to expose the *vena cava* and the *portal vein*. The portal vein was cannulated and liver perfusion medium (8 ml/min; Gibco/Thermo Fisher) was injected, followed by digestion medium (collagenase activity 200 U/ml, collagenase type II dissolved in DMEM medium) for 3–5 min. Ligaments were cut and the liver was transferred in 10 ml HC wash buffer (Gibco/Thermo Fisher). After removing the gall bladder, liver cells were released and subjected to a 70 µm cell strainer (Falcon). After adding 12 ml HC wash buffer, cells were centrifuged three times at 50 g at 4 °C for three minutes. The cell pellet contained HCs, while NPCs were enriched in the supernatant.

The HC pellet was resuspended in 20 ml Percoll solution after washed with HC wash buffer twice (GE Healthcare, Percoll/PBS, 2:3) and centrifuged with 50 g for 10 min to remove dead cells. Cells were resuspended in William E medium (Gibco/Thermo Fisher) for culture or in PBS with 2% FCS for counting and viability testing. Cell viability was visually determined by Trypan Blue staining.

NPCs in the supernatant were centrifuged at 500 g at 4 °C for 8 min. Cells were resuspended in 2 ml red blood cell (RBC) lysis buffer (Thermo Fisher) and immediately centrifuged at 500 g for 5 min. After washing with 5 ml PFB solution (2% FCS in PBS), NPCs were centrifuged at 500 g for 5 min, and the cell pellet was resuspended in 5 ml gradient buffer (2.5 ml PFB solution, 2.5 ml 40% iodixanol) followed by carefully adding 2 ml PFB on top. After centrifuging at 1,500 g (without brake) for 25 min, NPCs forming a ‘ring’ between the gradient layers were taken up in 8 ml PFB solution. After centrifugation at 500 g for 5 min, the cell pellet was resuspended in 6 ml separation buffer (1.2 ml PFB (without EDTA) and 4.8 ml 30% Histodenz, Sigma-Aldrich/Merck KGaA, Darmstadt, Germany) with 2 ml PBS on top. Cell suspension was centrifuged at 1,500 g without brake to remove cell debris for 23 min. Viable NPCs were enriched between the interface of PBS and the separation buffer. Cells were aspirated and resuspended in PFB solution followed by centrifugation at 500 g for 5 min. The cell pellet was resuspended with PFB solution and immediately used for viability testing. Cell viability was visually determined by Trypan Blue staining. Finally, viable and purified HCs and NPCs were mixed with a ratio of 3:7 and subjected to scRNA-seq analysis.

### scRNA-seq and data processing

Cell numbers were determined by counting using the brightfield option on the Auto Cell Counter (Logos Biosystems, Villeneuve d’Ascq, France). Following this, 16,000 cells were loaded onto the Chromium (10 × Genomics, Leiden, Netherlands) using the Chromium Next GEM Single Cell 3’ kit v3.1 (10 × Genomics). The single-cell suspension was processed as described in the manufacturer’s protocol. After cDNA synthesis, the samples were quantified with the Qubit High Sensitivity Assay (Invitrogen/Thermo Fisher). They were diluted for a quality check on the Agilent Bioanalyzer using a High Sensitivity Assay (Agilent Biotechnologies, Waldbronn, Germany). Libraries were then generated for NGS sequencing. Sequencing was performed at Novogene on the Illumina NovaSeq with 150 PE read output (Illumina, San Diego, USA). Sequence data were applied to Cell Ranger (version 6.0.1, 10X Genomics) to align reads and to generate the barcode unique molecular identifier (UMI) gene expression matrices based on the mouse reference genome mm10 with default parameters. scRNA-seq data have been deposited to the GEO database (GSE234260; https://www.ncbi.nlm.nih.gov/geo/).

### Unsupervised cell clustering, visualization, cell type identification, and expression analysis

The R package Seurat (v4.1.0) was used to perform the unsupervised clustering of cells based on the merged matrix of mouse data. Cells with < 500 or > 6,000 transcripts and mitochondrial gene content > 10% were excluded from further analysis.

The gene expression matrices were normalized to the total UMI counts per cell, followed by log transformation. The top 2,000 *highly variable genes* (HVGs) from the corrected expression matrix were obtained using the FindVariableFeatures function. After regressing cell cycle (S and G2/M scores calculated by the CellCycleScoring function in Seurat, principle component analysis (PCA) was performed to reduce dimensionality. Batch effects were corrected using the RunHarmony function with default parameters. The Louvain–Jaccard graph-based method identified k-nearest neighbor graphs (k-20) of main cell clusters. Next, the RunTSNE function with dimension parameters (1:20) in Seurat was used to reduce high-dimension data into two dimensions for visualization. Finally, the specifically expressed genes in each cell cluster were detected by the Seurat FindAllMarkers function using default parameters. The significant differences in gene expression were defined using the Wilcoxon rank-sum test with Bonferroni correction. The steps mentioned above were repeated for sub-clustering of all major cell types (endothelial cells: ECs, hepatocytes: HCs, Kupffer cells: KCs, hepatic stellate cell: HSCs, Macrophages: MACs, T cells: T, B cells: B, natural killer cells: NK, dendritic cells: DCs). For clustering all cells, EC, HC, KC, and MAC, the top 20, 15, 15, 15, and 20 PCs were selected with a resolution parameter equal to 0.4, 0.3, 0.2, 0.3, and 0.3, respectively.

Differentially expressed genes (DEGs) between two cell types were determined using the Wilcoxon rank-sum test with Bonferroni correction. DEGs were selected based on the following criteria: (1) expressed in more than 10% of the cells within either or both two groups; (2) |log_2_FC|≥ 0.25; 3) Wilcoxon rank-sum test adjusted *p*-value ≤ 0.05.

Gene Ontology Pathway enrichment analyses were performed with DEGs as the input gene list to gain insight into the biological functions of different cell clusters. Enrichment scores (p-values) were calculated using the clusterProfiler (v3.14.3) R package with a hyper-geometrical statistical test (threshold = 0.05). The Benjamini–Hochberg method was used to estimate the false discovery rate (FDR).

### ChIP-Seq data analysis

TEAD4 ChIP-Seq data from liver cancer cells (HepG2) were retrieved from GEO (GSE170161) [46, 47]. TEAD1 and TEAD4 Chip-Seq data from a cholangiocarcinoma cell line (HuCCT1) were retrieved from GEO (GSE68296) [70]. To visualize the ChIP-Seq data, BigWig files were binned, and the corresponding ChIP-Seq tracks were plotted using the function trackplot [71].

### Statistical analysis

Data are represented as mean -/ + standard deviation (SD) or Standard Error of Mean (SEM). Statistical tests used and sample sizes are described in Figure Legends. All statistical tests and graph preparation were done with Prism 9 software (GraphPad). Significance levels are as follows: *p ≤ 0.05, **p ≤ 0.01, ***p ≤ 0.001.

### Supplementary Information

Below is the link to the electronic supplementary material.

Supplementary file1 (TIFF 1966 KB) Supplementary Figure S1: CCl4-induced fibrosis and generation of YAP/TAZ-deficient mice. (A.) Exemplary sirius red stain of WT mice after CCl4 treatment (2x for six weeks followed by four weeks without injection) illustrates mild peri-portal and porto-portal bridging fibrosis. (B.) Real-time PCR of murine YAP and TAZ in liver lysates from WT (n=8), YAP^KO^ (n=8), TAZ^KO^ (n=8), and DKO (n=9) male mice. For normalization, the expression of murine GAPDH and PPIA were used. (C.) Exemplary Western immunoblot of whole liver tissue lysates. Next to WT mice (n=2), animals with heterozygous YAP deletion and homozygous TAZ deletions (YAP^+/Δ^/TAZ^Δ/Δ^; n=2), homozygous YAP deletions and heterozygous TAZ deletion (YAP^Δ/Δ^/TAZ^+/Δ^; n=2) are shown. (D.) A machine learning algorithm was used to quantify YAP and TAZ positivity in HCs. YAP-positive NPCs in YAP^KO^ and DKO samples were detected and excluded from further analysis (Figure 3). Graphs show results from up to 404 tiles per mouse line. Data illustrate diminished YAP levels in YAP^KO^ and DKO mice. TAZ levels in TAZ^KO^ and DKO mice appear to be unchanged as TAZ is not efficiently detected by immunohistochemistry in HCs and BECs. (E.) Immunohistochemical staining of the YAP target gene CTGF. Black arrowheads: bile ducts with BECs. (F.) Average necrosis diameter in YAP^KO^ (n=13 with 142 lesions) and DKO mice (n=8 with 82 lesions). Statistical test: Mann–Whitney U test. ***p≤0.001. (G.) Liver/body weight ratio of WT (n=9), YAP^KO^ (n=7), TAZ^KO^ (n=6), and DKO (n=7) female mice. Statistical test: ANOVA with Dunnett’s multiple comparisons test. ns: not significant. (H.) Serum liver damage markers ALT/AST in female WT (n=5/4), YAP^KO^ (n=7/7), TAZ^KO^ (n=5/4), and DKO (n=7/5) animals. Statistical test: Dunnett’s multiple comparisons. *p≤0.05, ns: not significant. For G./H.: Statistical comparisons not displayed do not reach the significance level (p>0.05).

Supplementary file2 (TIFF 512 KB) Supplementary Figure S2: Detection of YAP/TEAD and TAZ/TEAD interaction in HC-derived cells. Human HLF cells were used as a model system for coIP experiments. (A.) Western immunoblotting revealed that transfection of YAP- and TAZ-specific siRNAs efficiently diminished both proteins after 48 hr. (B.) Western immunoblot after immunoprecipitation (IP) of TEAD family members. YAP and TAZ antibodies detected both proteins bound to TEADs. The membrane was cut around 60 kDa for YAP or TAZ antibody incubation. (C.) Western immunoblot after IP of YAP or TAZ. A pan-TEAD antibody detected TEAD family members bound to YAP or TAZ. NTC: no template control. siYAP/siTAZ: transfection of gene-specific siRNA (20 nM). For input, 10% of the protein fraction was used. IP with IgG but without protein fraction served as control.

Supplementary file3 (TIFF 6789 KB) Supplementary Figure S3: Comparison of YAP positivity in EC cells. Exemplary YAP and TAZ stains in the portal field, parenchyma, and central vein are shown. Black arrows: YAP- and TAZ-negative “big vessel” ECs in WT, YAP^KO^, TAZ^KO^, and DKO mice. Red arrows: YAP-positive “big vessel” ECs. Red arrowheads: positive sinusoidal ECs.

Supplementary file4 (TIFF 1448 KB) Supplementary Figure S4: Scheme summarizing the single-cell isolation protocol from murine liver tissues. One mouse from each mouse strain (WT, YAP^KO^, TAZ^KO^, and DKO) was subjected to single-cell isolation. NPCs and HCs were isolated separately to obtain the highest quality of viability cell populations. Immediately after isolating viable NPC and HC populations, both cell fractions were mixed (ratio 7:3) and subjected to scRNA-seq analysis. The figure was modified using Servier Medical Art (http://smart.servier.com).

Supplementary file5 (TIFF 370 KB) Supplementary Figure S5: Marker panel used for the identification of hepatic cells. Bubble plot showing the expression of marker genes for different hepatic cell types. Marker profiles used for the identification of cell clusters were: VTN, AGXT, RGN (HCs); SPP1, SOX9, KRT19 (BECs); PECAM1, NTN4, EFNB1, CDH13 (ECs); NKG7, KLRE1, XCL1 (NK cells); TRBC2, CD3D, CD3G (T cells); CD79A, EBF1, PAX5 (B cells); CLEC4F, VISIG4, TIMD4 (KCs); LYZ2, CX3CR1, ITGAM (MACs); CD74, IRF8, SIGLECH, CD300C (DC cluster 2); DCN, COLEC11, RELN (HSCs). Although separated in the t-SNE plot, DC cluster 1 very likely belongs to the MACs group as similar biomarkers are expressed.

Supplementary file6 (TIFF 6157 KB) Supplementary Figure S6: Heatmap showing the top ten genes discriminating EC subclusters. The colored bars indicate subclusters EC-0 to EC-7. The top ten genes characterizing each cluster are shown on the left side.

Supplementary file7 (TIFF 1205 KB) Supplementary Figure S7: Subclusters without induction of YAP in ECs and KCs. (A.) Violin plot showing variable YAP expression in the subclusters EC-0, EC-1, EC-3, EC-4, EC-5, and EC-6 isolated from WT, YAP^KO^, TAZ^KO^ and DKO mice. (B.) Violin plot showing variable YAP expression in the subclusters KC-0, KC-2, and KC-3 in tissue samples derived from WT, YAP^KO^, TAZ^KO^, and DKO mice.

Supplementary file8 (TIFF 5952 KB) Supplementary Figure S8: Heatmap showing the top ten genes discriminating KC subclusters.The colored bars indicate subclusters KC-0 to EC-4. The top ten genes characterizing respective clusters are shown on the left side.

Supplementary file9 (TIFF 5227 KB) Supplementary Figure S9: HSC subpopulations in mouse livers of WT, YAP^KO^, TAZ^KO^, and DKO mice. (A.) Identification of HSC subclusters. Three HSC clusters were identified (HSC-0, HSC-1, and HSC-2). Further unsupervised clustering of HSC-2 revealed four HSC groups with distinct molecular features (HSC-2/0 to HSC-2/3). For example, HSC-2/0 cells showed characteristics of HGF-positive cyHSCs, while COL1A1 was enriched in HSC-2/3 and defined myHSCs. (B.) Heatmap of top expressed genes in subpopulations HSC-2/0, HSC-2/1, HSC-2/2, and HSC-2/3. (C.) Thirteen of fourteen genes defining the subpopulation HSC-2/0 have been described as cyHSC-specific signature genes (see Filiol et al., 2022, Nature). Violin plots illustrate that YAP mRNA is not differentially expressed in the investigated mouse lines. Results for HSC-0 (D.), HSC-1 (E.), and HSC-2 (F.) are shown.

Supplementary file10 (TIFF 655 KB) Supplementary Figure S10: Expression of genes in EC and KC subclusters. (A.) Violin plots illustrate elevated transcript levels of the chemokines/cytokines CXCL10, IL1, and CXCL2 in YAP-positive subgroups EC-2 and EC-7. (B.) Violin plots illustrate elevated transcript levels of the S100 family members S100A6, S100A8, and S100A9 in the YAP-positive EC subgroup EC-7.

Supplementary file11 (TIFF 2281 KB) Supplementary Figure S11: Schematic display of image analysis algorithm used for signal quantification. (A.) Stained tissues are digitalized at high magnification (40x) and divided into tiles (1 mm^2^). (B.) Tiles with artifacts or low viable tissue content are excluded. (C.) Random forest machine learning algorithm is applied on the tile level using Ilastik software. Random forest combined the output of multiple decision trees to detect the highest scoring class for each tile pixel. (D.) Based on the classification result, tissue structures are detected, such as nuclei in areas with specific staining (here exemplified as a red overlay for YAP positivity in HCs).

Supplementary file12 (TIFF 13180 KB) Supplementary Figure S12: Characterization of immune response in WT, YAP^KO^, TAZ^KO^, and DKO livers. Immunohistochemical staining of the granulocyte marker MPO (A.), the T cell marker CD8 (B.), antigen-presenting MHC-II (C.), the T cell markers CD3 and CD4 (D./E.), and the B cell marker B220 (F.). Violin plots illustrate respective quantification. WT (n=4), YAP^KO^ (n=6), TAZ^KO^ (n=4), and DKO (n=6) animals were analyzed using a machine learning algorithm. Statistical test: ANOVA with Dunnett’s multiple comparisons test. *p≤0.05, ns: not significant. In total, 1.343 (MPO), 1.468 (MHCII), 906 (CD3), 1.121 (CD4), and 1.250 (B220) tiles were quantitatively investigated. Statistical comparisons not displayed do not reach the significance level (p>0.05).

Supplementary file13 (TIFF 1655 KB) Supplementary Figure S13: Effects of CXCL11 and FLT3L on cells of myeloid and endothelial origin. (A.)Western immunoblot of protein fractions isolated from the endothelial cell line SVEC4-10 after treatment with CXCL11 and FLT3L. Signal quantification and normalization with actin showed a 10-20% induction of YAP after FLT3L and CXCL11 treatment, respectively. (B.) Immunofluorescence analysis of myeloid RAW264 cells after CXCL11 and FLT3L treatment demonstrated elevated YAP levels. YAP intensity and the cell number were quantitatively measured. (C.) Lateral migration of an immortalized murine KC cell line. Cells were treated with CXCL11 or FLT3L for 48 hrs. Gaps were digitally documented and cell-free areas were quantified. Nine (0 hrs) and ten (ctrl, CXCL11, and FLT3L after 48 hrs) images were analyzed. Statistical test: ANOVA with Dunnett’s multiple comparisons test. **p≤0.01, ***p≤0.001, ns: not significant. ctrl: control.

Supplementary file14 (DOCX 19 KB): Supplementary Tables S1 and S2

## Data Availability

scRNA-seq data have been deposited to the GEO database (GSE234260; https://www.ncbi.nlm.nih.gov/geo/).

## Abbreviations

BDL: Bile duct ligation
BEC: Biliary epithelial cell
CCl_4_: Carbon tetrachloride
CXCL11: C-X-C motif chemokine ligand 11
DKO: Double knock-out
EC: Endothelial cell
FLT3L: fms-related receptor tyrosine kinase 3 ligand
HCC: Hepatocellular carcinoma
HC: Hepatocyte
HSC: Hepatic stellate cell
ICAM1: Intercellular adhesion molecule-1
IL1: Interleukin-1
KC: Kupffer cell
LSEC: Liver sinusoidal endothelial cell
MDR2: Multidrug resistance 2
NPC: Non-parenchymal cell
PHx: Partial hepatectomy
TAZ: Transcriptional coactivator with PDZ-binding motif
TEAD: TEA domain transcription factor
YAP: Yes-associated protein
