# Supplementary figures and images for: Dynamic YAP expression in the non-parenchymal liver cell compartment controls heterologous cell communication
Source: Cell Mol Life Sci. 2024 Mar 4;81(1):115. doi: 10.1007/s00018-024-05126-1 (PMC10912141; doi:10.1007/s00018-024-05126-1)

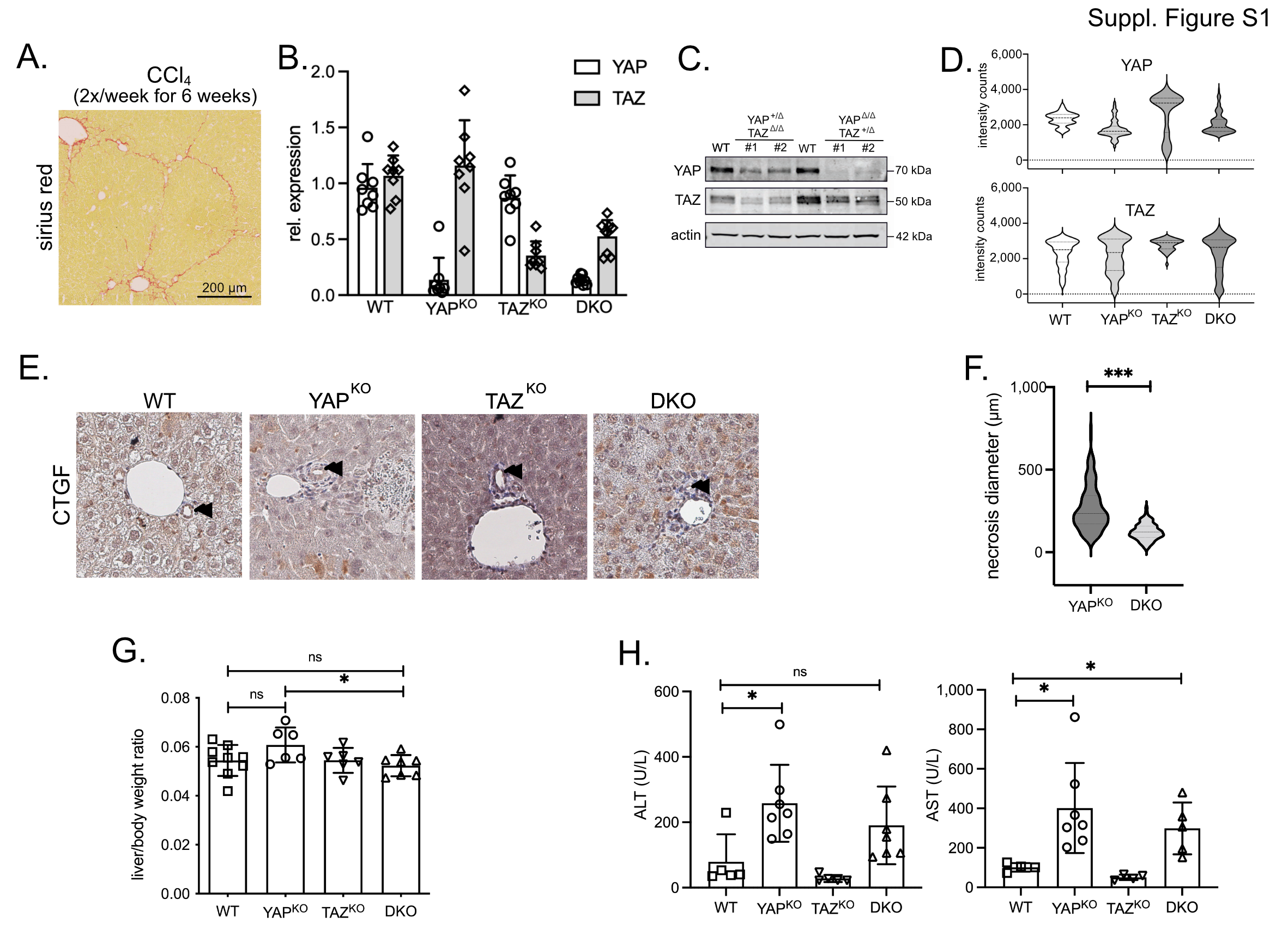

Supplement: Supplementary file 1 — Supplementary file1 (TIFF 1966 KB) Supplementary Figure S1: CCl4-induced fibrosis and generation of YAP/TAZ-deficient mice. (A.) Exemplary sirius red stain of WT mice after CCl4 treatment (2x for six weeks followed by four weeks without injection) illustrates mild peri-portal and porto-portal bridging fibrosis. (B.) Real-time PCR of murine YAP and TAZ in liver lysates from WT (n=8), YAPKO (n=8), TAZKO (n=8), and DKO (n=9) male mice. For normalization, the expression of murine GAPDH and PPIA were used. (C.) Exemplary Western immunoblot of whole liver tissue lysates. Next to WT mice (n=2), animals with heterozygous YAP deletion and homozygous TAZ deletions (YAP+/Δ/TAZΔ/Δ; n=2), homozygous YAP deletions and heterozygous TAZ deletion (YAPΔ/Δ/TAZ+/Δ; n=2) are shown. (D.) A machine learning algorithm was used to quantify YAP and TAZ positivity in HCs. YAP-positive NPCs in YAPKO and DKO samples were detected and excluded from further analysis (Figure 3). Graphs show results from up to 404 tiles per mouse line. Data illustrate diminished YAP levels in YAPKO and DKO mice. TAZ levels in TAZKO and DKO mice appear to be unchanged as TAZ is not efficiently detected by immunohistochemistry in HCs and BECs. (E.) Immunohistochemical staining of the YAP target gene CTGF. Black arrowheads: bile ducts with BECs. (F.) Average necrosis diameter in YAPKO (n=13 with 142 lesions) and DKO mice (n=8 with 82 lesions). Statistical test: Mann–Whitney U test. ***p≤0.001. (G.) Liver/body weight ratio of WT (n=9), YAPKO (n=7), TAZKO (n=6), and DKO (n=7) female mice. Statistical test: ANOVA with Dunnett’s multiple comparisons test. ns: not significant. (H.) Serum liver damage markers ALT/AST in female WT (n=5/4), YAPKO (n=7/7), TAZKO (n=5/4), and DKO (n=7/5) animals. Statistical test: Dunnett’s multiple comparisons. *p≤0.05, ns: not significant. For G./H.: Statistical comparisons not displayed do not reach the significance level (p>0.05). [file 18_2024_5126_MOESM1_ESM.tiff]

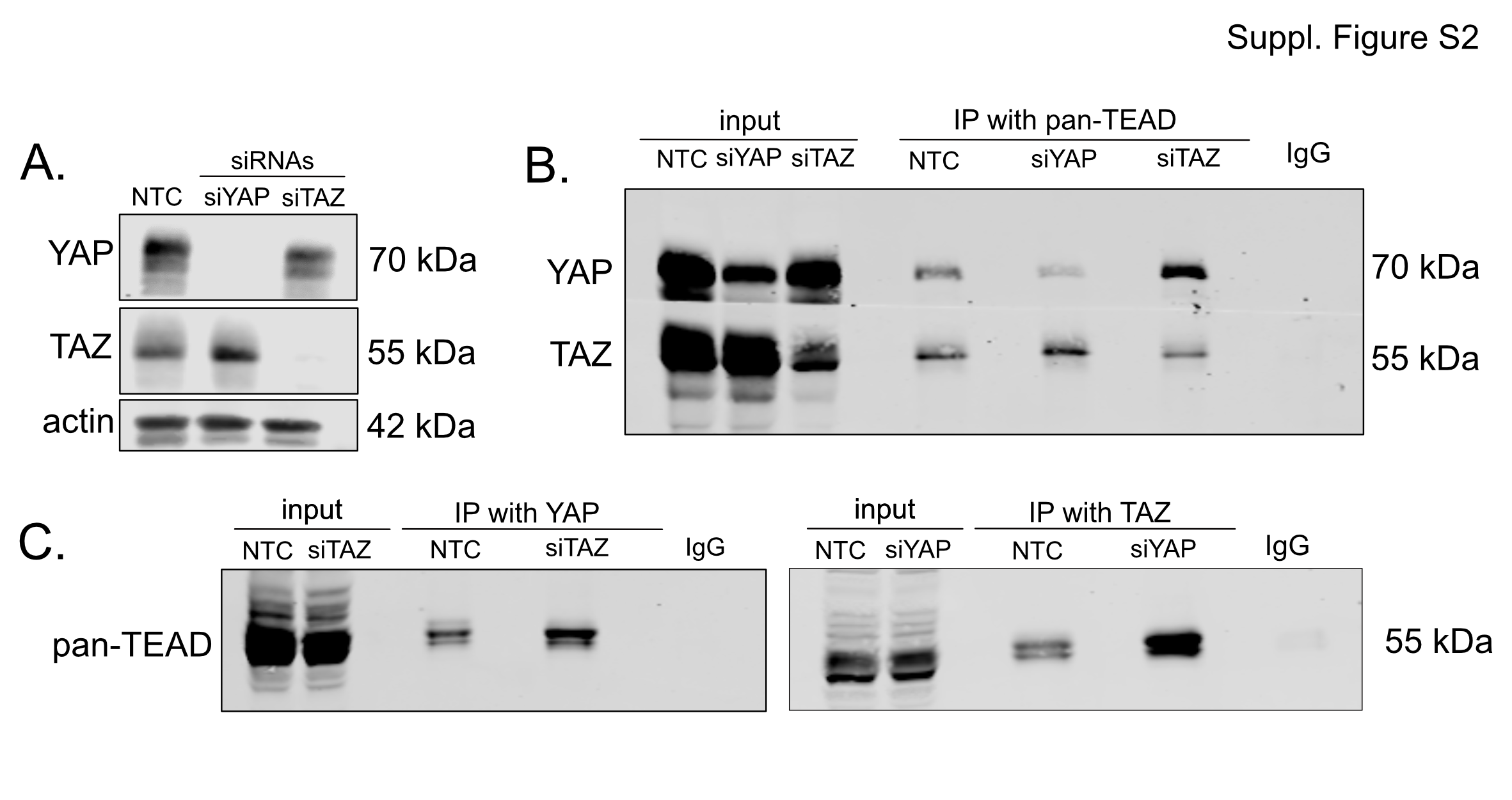

Supplement: Supplementary file 2 — Supplementary file2 (TIFF 512 KB) Supplementary Figure S2: Detection of YAP/TEAD and TAZ/TEAD interaction in HC-derived cells. Human HLF cells were used as a model system for coIP experiments. (A.) Western immunoblotting revealed that transfection of YAP- and TAZ-specific siRNAs efficiently diminished both proteins after 48 hr. (B.) Western immunoblot after immunoprecipitation (IP) of TEAD family members. YAP and TAZ antibodies detected both proteins bound to TEADs. The membrane was cut around 60 kDa for YAP or TAZ antibody incubation. (C.) Western immunoblot after IP of YAP or TAZ. A pan-TEAD antibody detected TEAD family members bound to YAP or TAZ. NTC: no template control. siYAP/siTAZ: transfection of gene-specific siRNA (20 nM). For input, 10% of the protein fraction was used. IP with IgG but without protein fraction served as control. [file 18_2024_5126_MOESM2_ESM.tiff]

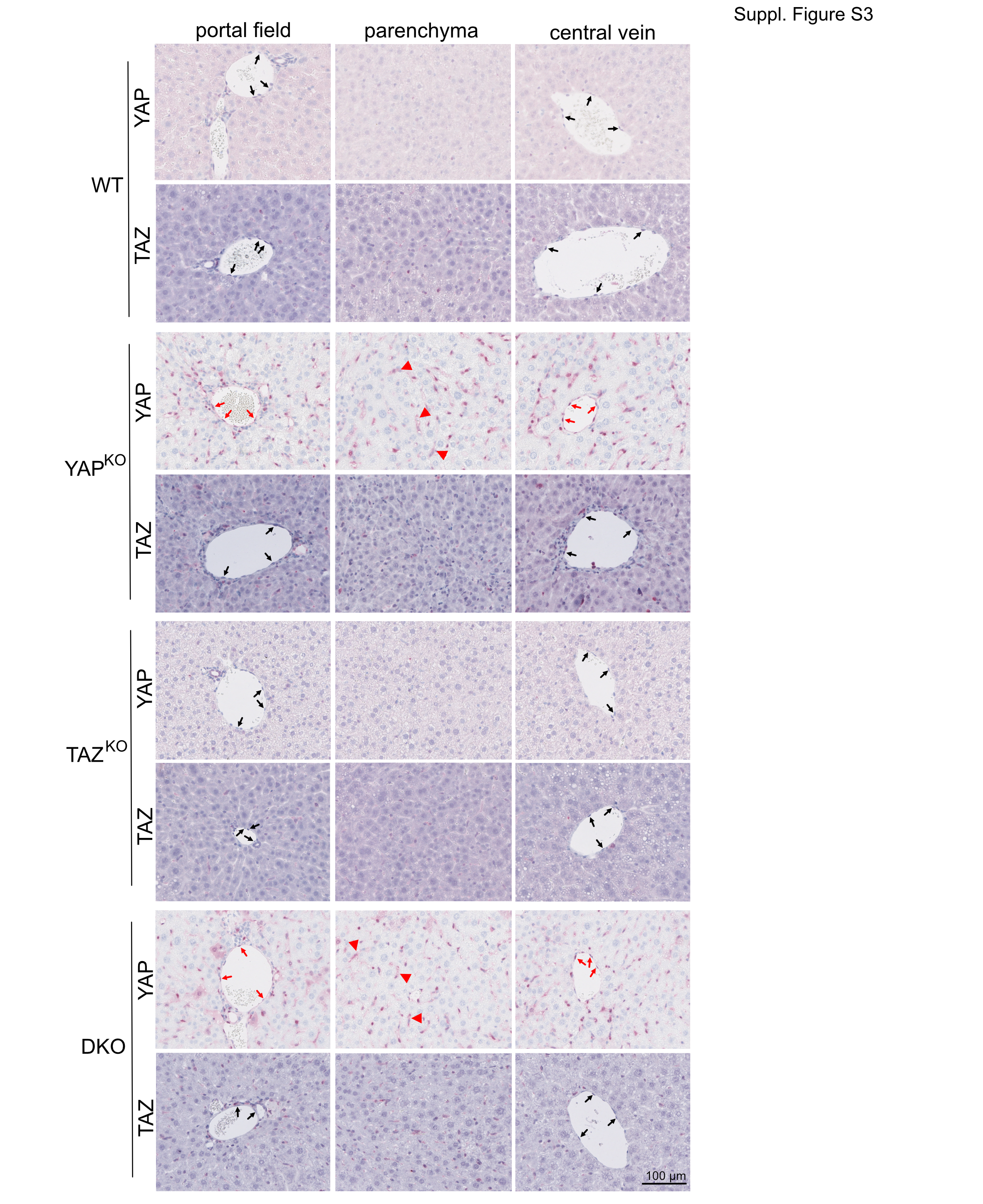

Supplement: Supplementary file 3 — Supplementary file3 (TIFF 6789 KB) Supplementary Figure S3: Comparison of YAP positivity in EC cells. Exemplary YAP and TAZ stains in the portal field, parenchyma, and central vein are shown. Black arrows: YAP- and TAZ-negative “big vessel” ECs in WT, YAPKO, TAZKO, and DKO mice. Red arrows: YAP-positive “big vessel” ECs. Red arrowheads: positive sinusoidal ECs. [file 18_2024_5126_MOESM3_ESM.tiff]

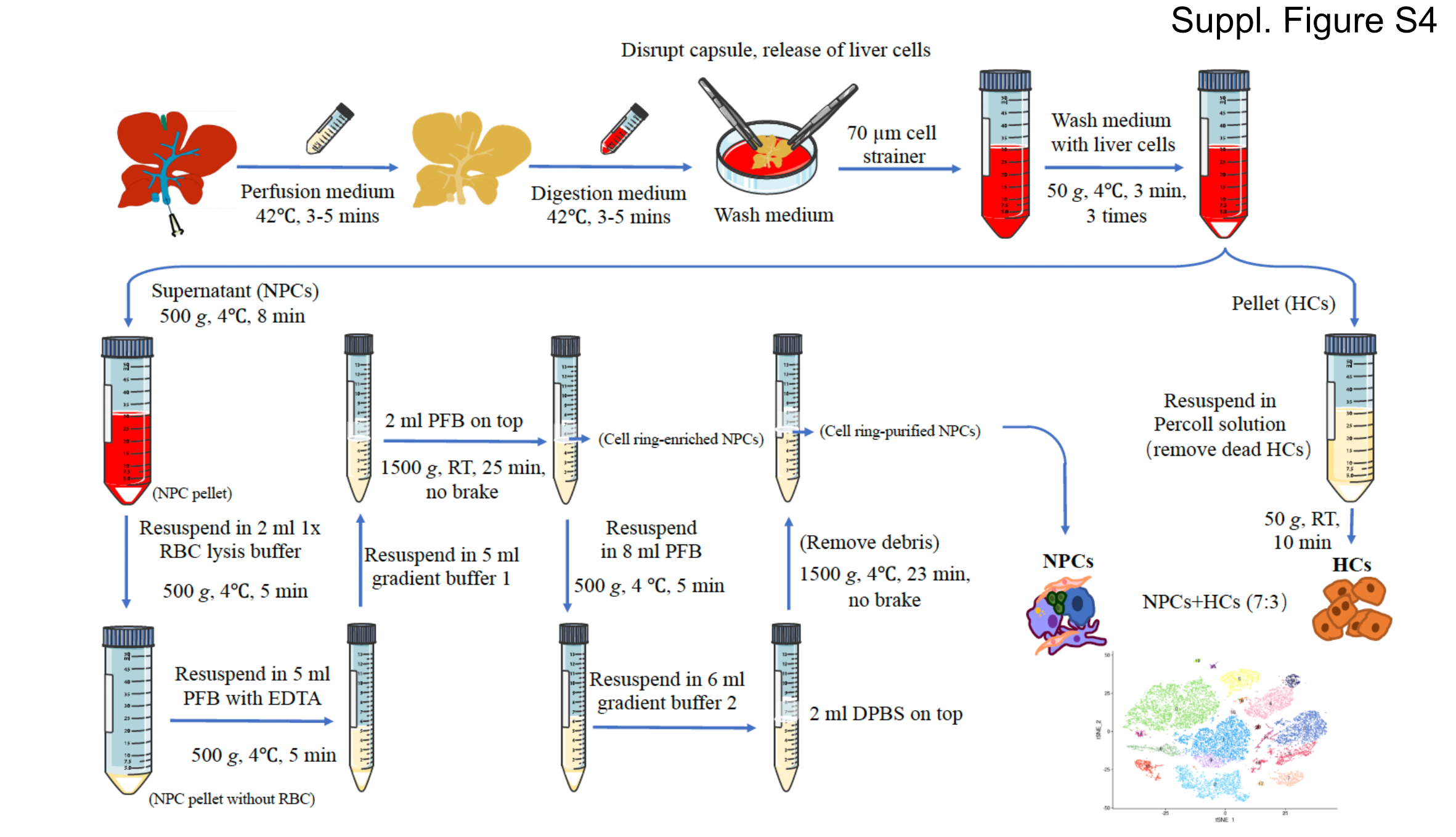

Supplement: Supplementary file 4 — Supplementary file4 (TIFF 1448 KB) Supplementary Figure S4: Scheme summarizing the single-cell isolation protocol from murine liver tissues. One mouse from each mouse strain (WT, YAPKO, TAZKO, and DKO) was subjected to single-cell isolation. NPCs and HCs were isolated separately to obtain the highest quality of viability cell populations. Immediately after isolating viable NPC and HC populations, both cell fractions were mixed (ratio 7:3) and subjected to scRNA-seq analysis. The figure was modified using Servier Medical Art (http://smart.servier.com). [file 18_2024_5126_MOESM4_ESM.tiff]

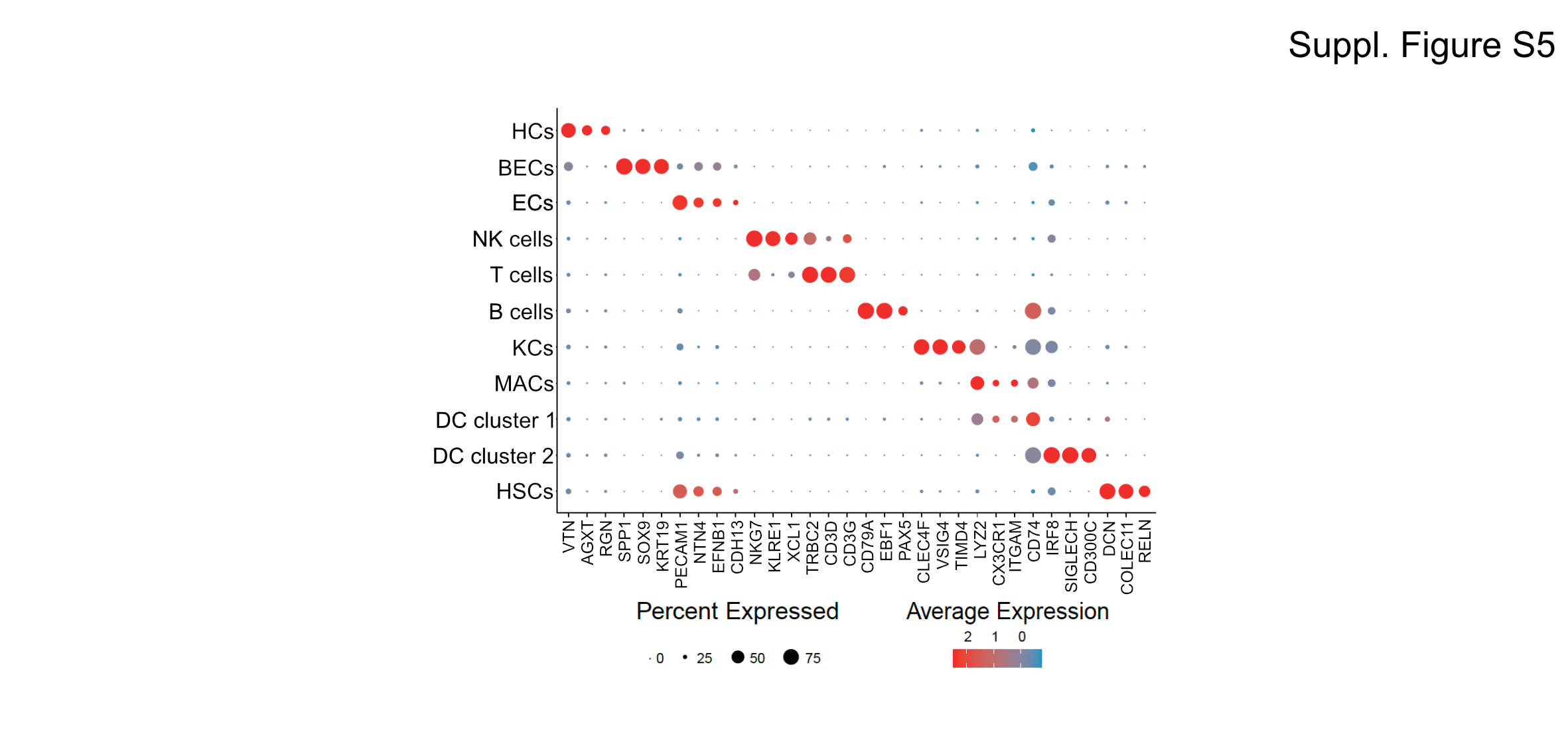

Supplement: Supplementary file 5 — Supplementary file5 (TIFF 370 KB) Supplementary Figure S5: Marker panel used for the identification of hepatic cells. Bubble plot showing the expression of marker genes for different hepatic cell types. Marker profiles used for the identification of cell clusters were: VTN, AGXT, RGN (HCs); SPP1, SOX9, KRT19 (BECs); PECAM1, NTN4, EFNB1, CDH13 (ECs); NKG7, KLRE1, XCL1 (NK cells); TRBC2, CD3D, CD3G (T cells); CD79A, EBF1, PAX5 (B cells); CLEC4F, VISIG4, TIMD4 (KCs); LYZ2, CX3CR1, ITGAM (MACs); CD74, IRF8, SIGLECH, CD300C (DC cluster 2); DCN, COLEC11, RELN (HSCs). Although separated in the t-SNE plot, DC cluster 1 very likely belongs to the MACs group as similar biomarkers are expressed. [file 18_2024_5126_MOESM5_ESM.tiff]

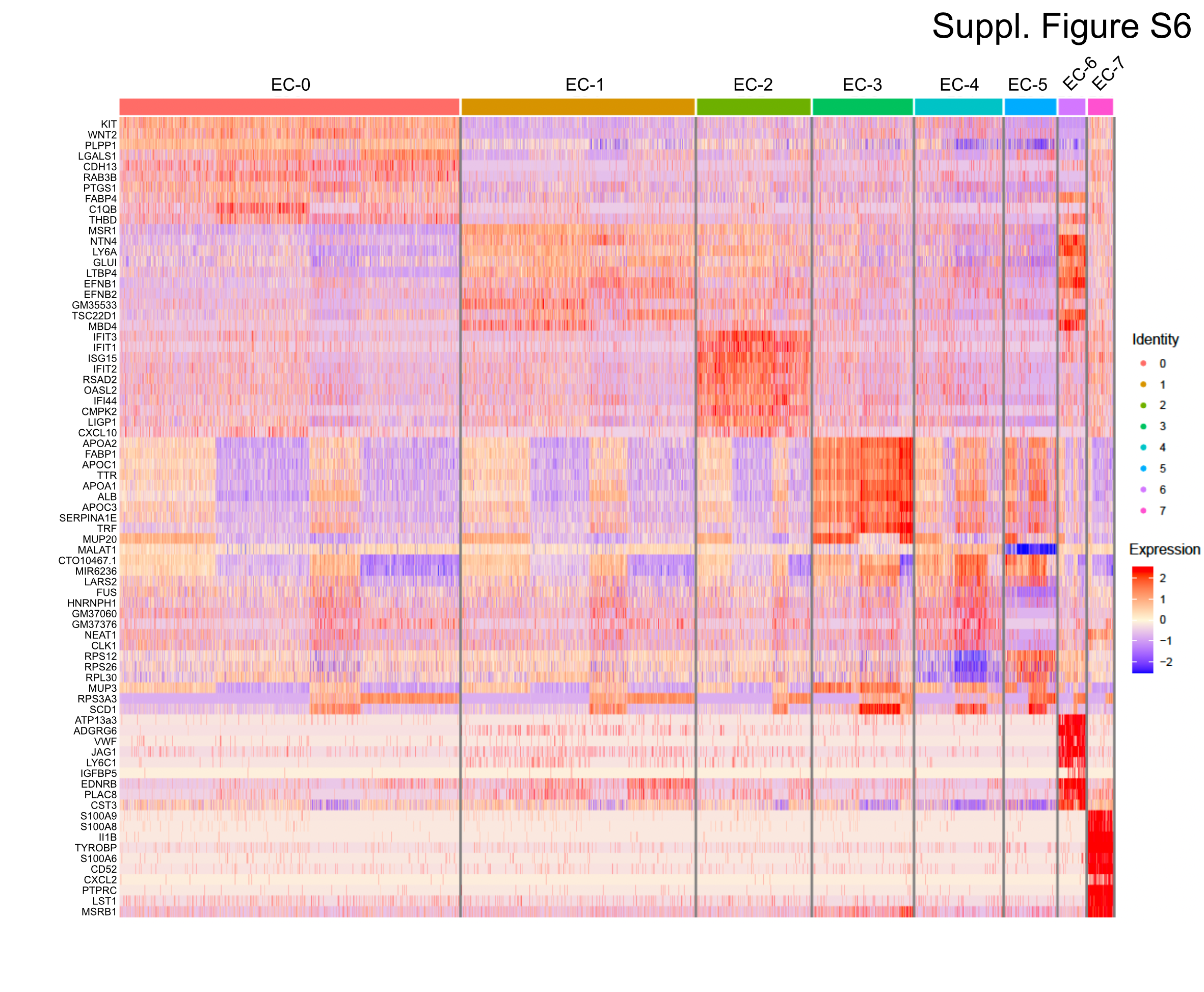

Supplement: Supplementary file 6 — Supplementary file6 (TIFF 6157 KB) Supplementary Figure S6: Heatmap showing the top ten genes discriminating EC subclusters. The colored bars indicate subclusters EC-0 to EC-7. The top ten genes characterizing each cluster are shown on the left side. [file 18_2024_5126_MOESM6_ESM.tiff]

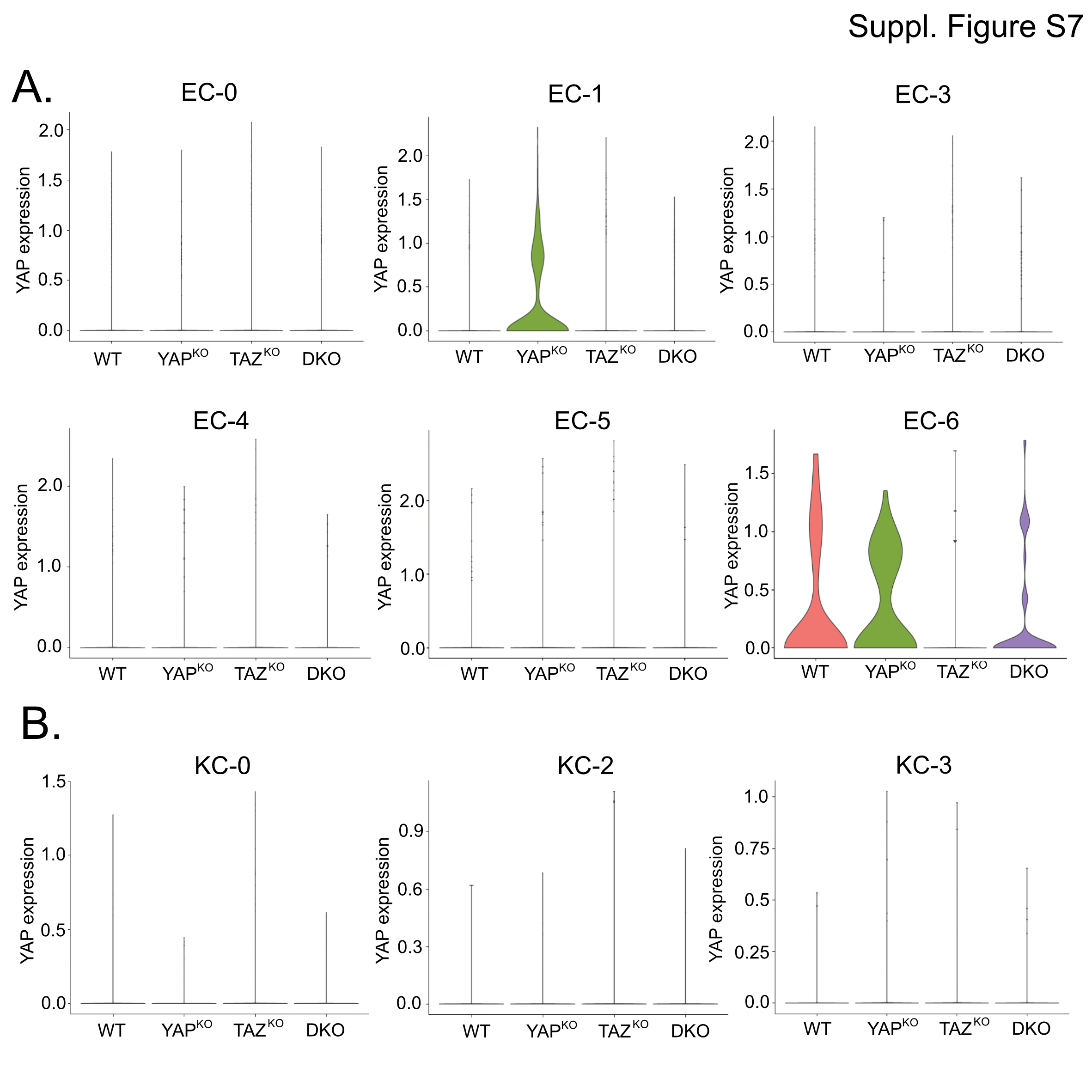

Supplement: Supplementary file 7 — Supplementary file7 (TIFF 1205 KB) Supplementary Figure S7: Subclusters without induction of YAP in ECs and KCs. (A.) Violin plot showing variable YAP expression in the subclusters EC-0, EC-1, EC-3, EC-4, EC-5, and EC-6 isolated from WT, YAPKO, TAZKO and DKO mice. (B.) Violin plot showing variable YAP expression in the subclusters KC-0, KC-2, and KC-3 in tissue samples derived from WT, YAPKO, TAZKO, and DKO mice. [file 18_2024_5126_MOESM7_ESM.tiff]

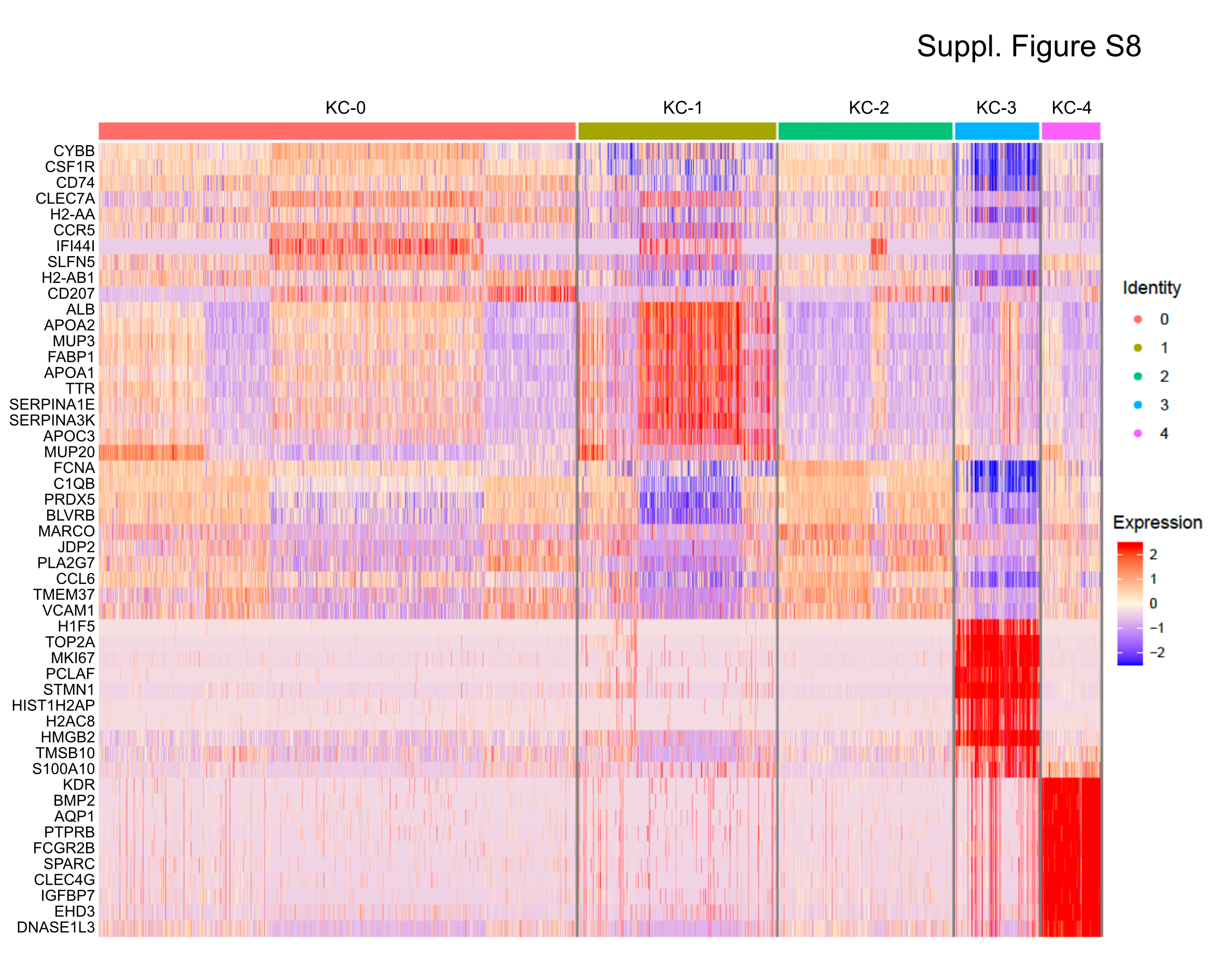

Supplement: Supplementary file 8 — Supplementary file8 (TIFF 5952 KB) Supplementary Figure S8: Heatmap showing the top ten genes discriminating KC subclusters.The colored bars indicate subclusters KC-0 to EC-4. The top ten genes characterizing respective clusters are shown on the left side. [file 18_2024_5126_MOESM8_ESM.tiff]

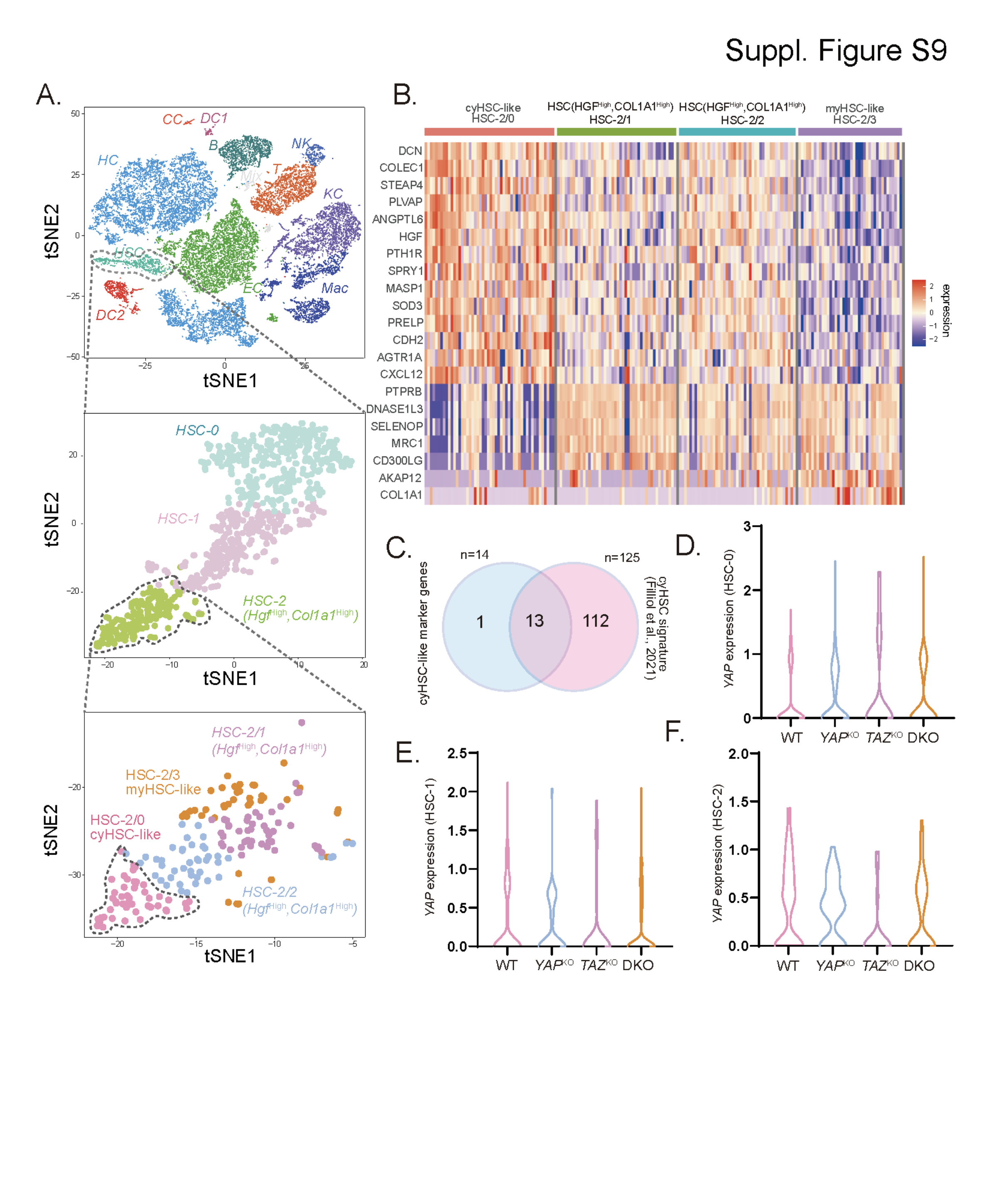

Supplement: Supplementary file 9 — Supplementary file9 (TIFF 5227 KB) Supplementary Figure S9: HSC subpopulations in mouse livers of WT, YAPKO, TAZKO, and DKO mice. (A.) Identification of HSC subclusters. Three HSC clusters were identified (HSC-0, HSC-1, and HSC-2). Further unsupervised clustering of HSC-2 revealed four HSC groups with distinct molecular features (HSC-2/0 to HSC-2/3). For example, HSC-2/0 cells showed characteristics of HGF-positive cyHSCs, while COL1A1 was enriched in HSC-2/3 and defined myHSCs. (B.) Heatmap of top expressed genes in subpopulations HSC-2/0, HSC-2/1, HSC-2/2, and HSC-2/3. (C.) Thirteen of fourteen genes defining the subpopulation HSC-2/0 have been described as cyHSC-specific signature genes (see Filiol et al., 2022, Nature). Violin plots illustrate that YAP mRNA is not differentially expressed in the investigated mouse lines. Results for HSC-0 (D.), HSC-1 (E.), and HSC-2 (F.) are shown. [file 18_2024_5126_MOESM9_ESM.tiff]

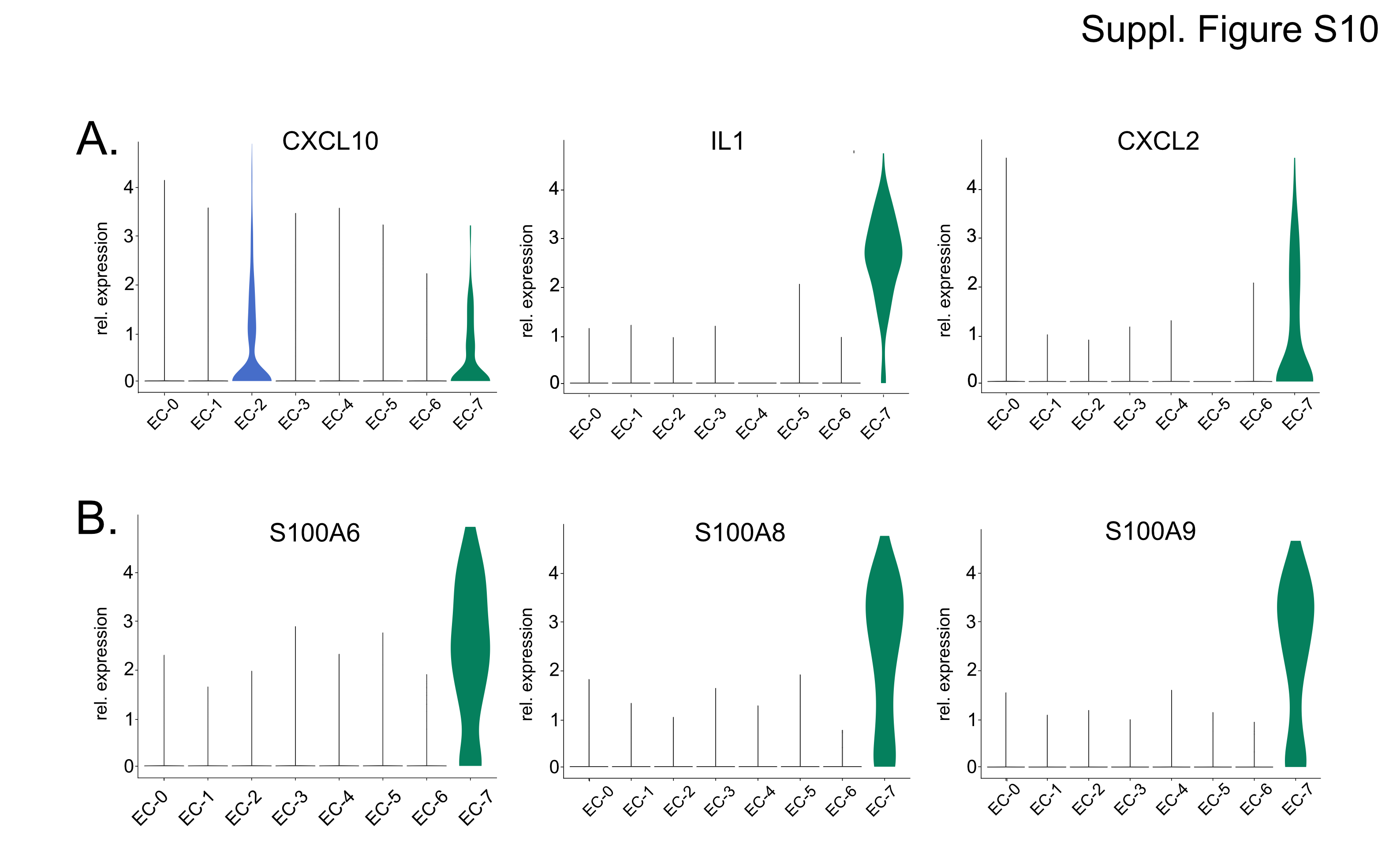

Supplement: Supplementary file 10 — Supplementary file10 (TIFF 655 KB) Supplementary Figure S10: Expression of genes in EC and KC subclusters. (A.) Violin plots illustrate elevated transcript levels of the chemokines/cytokines CXCL10, IL1, and CXCL2 in YAP-positive subgroups EC-2 and EC-7. (B.) Violin plots illustrate elevated transcript levels of the S100 family members S100A6, S100A8, and S100A9 in the YAP-positive EC subgroup EC-7. [file 18_2024_5126_MOESM10_ESM.tiff]

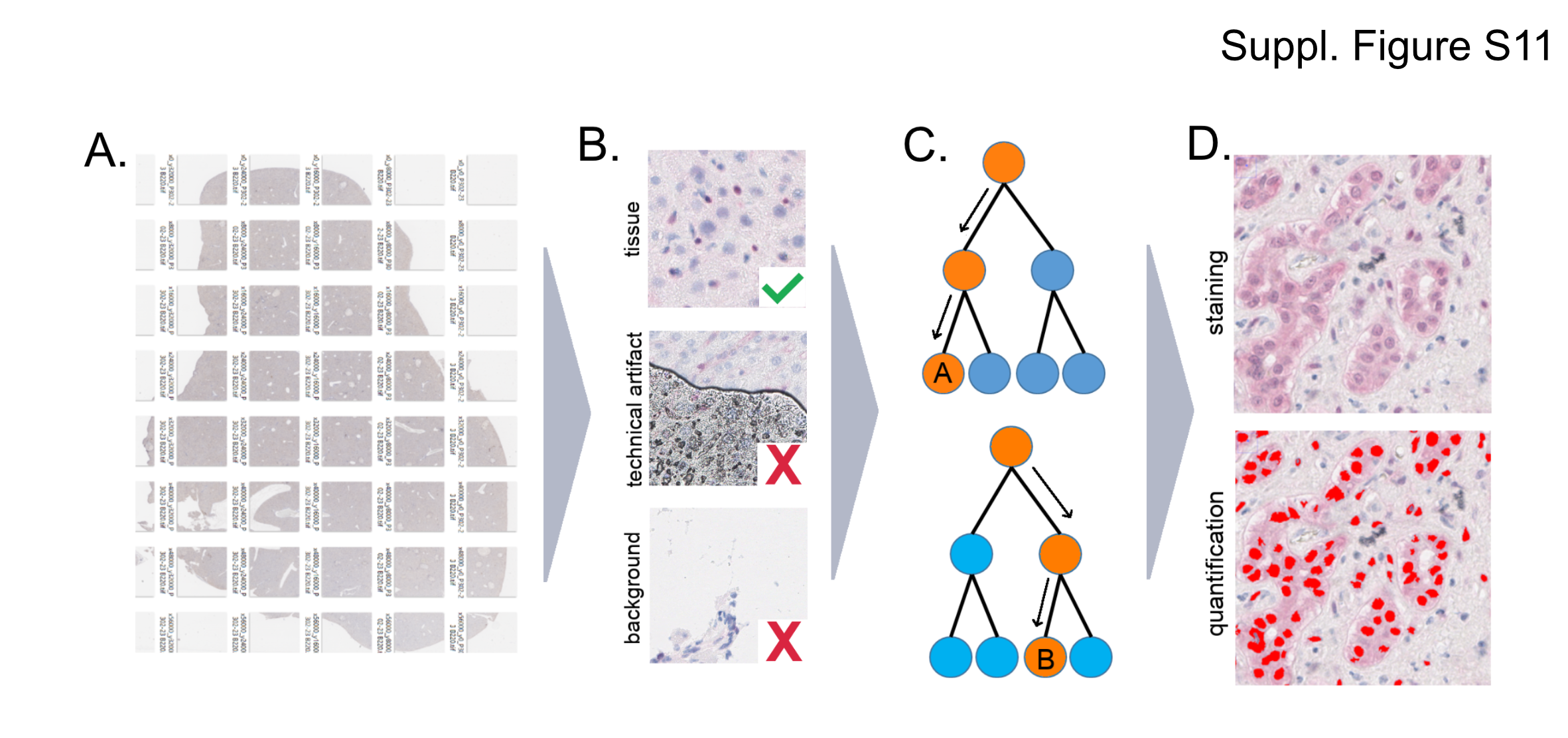

Supplement: Supplementary file 11 — Supplementary file11 (TIFF 2281 KB) Supplementary Figure S11: Schematic display of image analysis algorithm used for signal quantification. (A.) Stained tissues are digitalized at high magnification (40x) and divided into tiles (1 mm2). (B.) Tiles with artifacts or low viable tissue content are excluded. (C.) Random forest machine learning algorithm is applied on the tile level using Ilastik software. Random forest combined the output of multiple decision trees to detect the highest scoring class for each tile pixel. (D.) Based on the classification result, tissue structures are detected, such as nuclei in areas with specific staining (here exemplified as a red overlay for YAP positivity in HCs). [file 18_2024_5126_MOESM11_ESM.tiff]

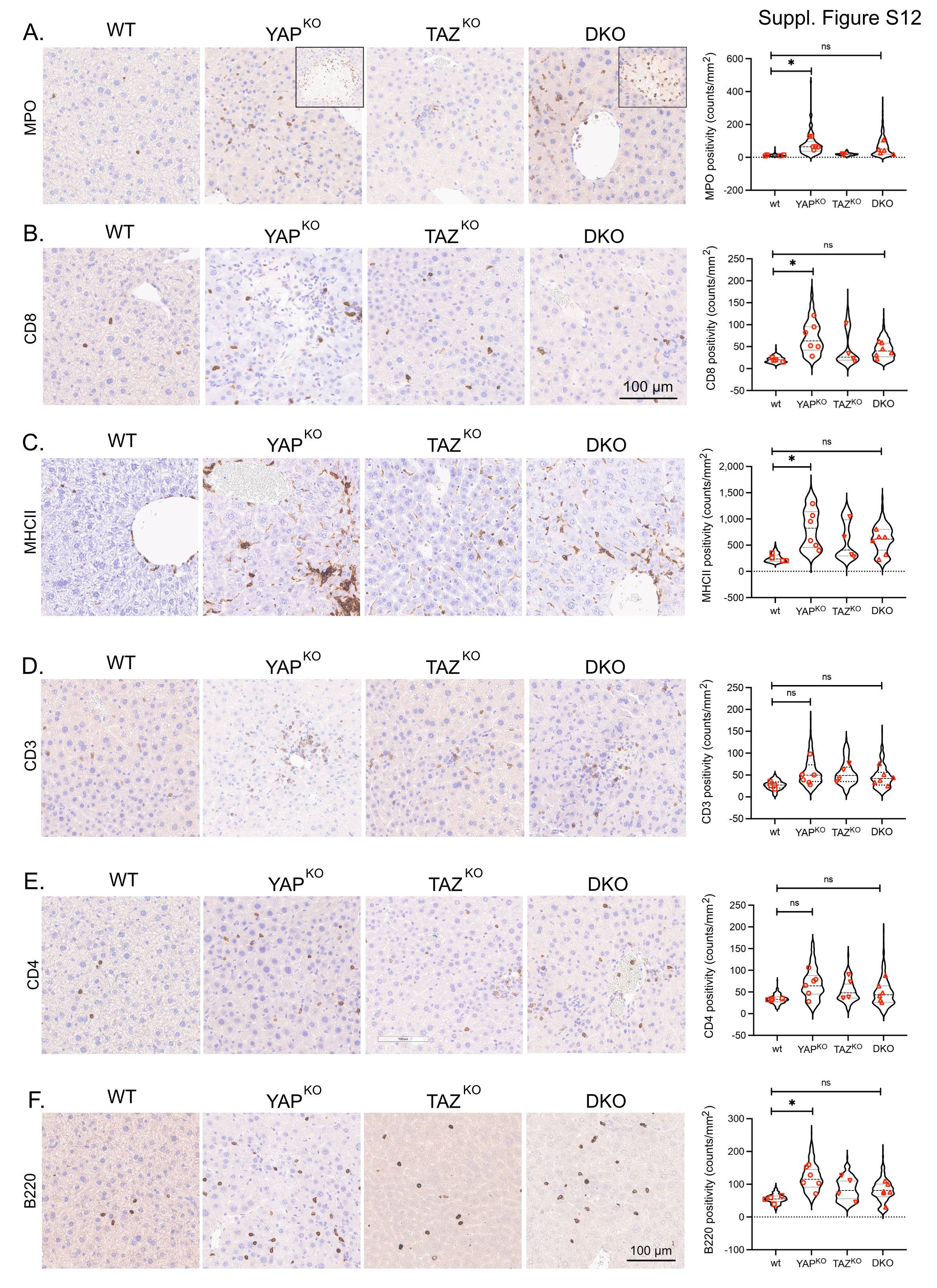

Supplement: Supplementary file 12 — Supplementary file12 (TIFF 13180 KB) Supplementary Figure S12: Characterization of immune response in WT, YAPKO, TAZKO, and DKO livers. Immunohistochemical staining of the granulocyte marker MPO (A.), the T cell marker CD8 (B.), antigen-presenting MHC-II (C.), the T cell markers CD3 and CD4 (D./E.), and the B cell marker B220 (F.). Violin plots illustrate respective quantification. WT (n=4), YAPKO (n=6), TAZKO (n=4), and DKO (n=6) animals were analyzed using a machine learning algorithm. Statistical test: ANOVA with Dunnett’s multiple comparisons test. *p≤0.05, ns: not significant. In total, 1.343 (MPO), 1.468 (MHCII), 906 (CD3), 1.121 (CD4), and 1.250 (B220) tiles were quantitatively investigated. Statistical comparisons not displayed do not reach the significance level (p>0.05). [file 18_2024_5126_MOESM12_ESM.tiff]

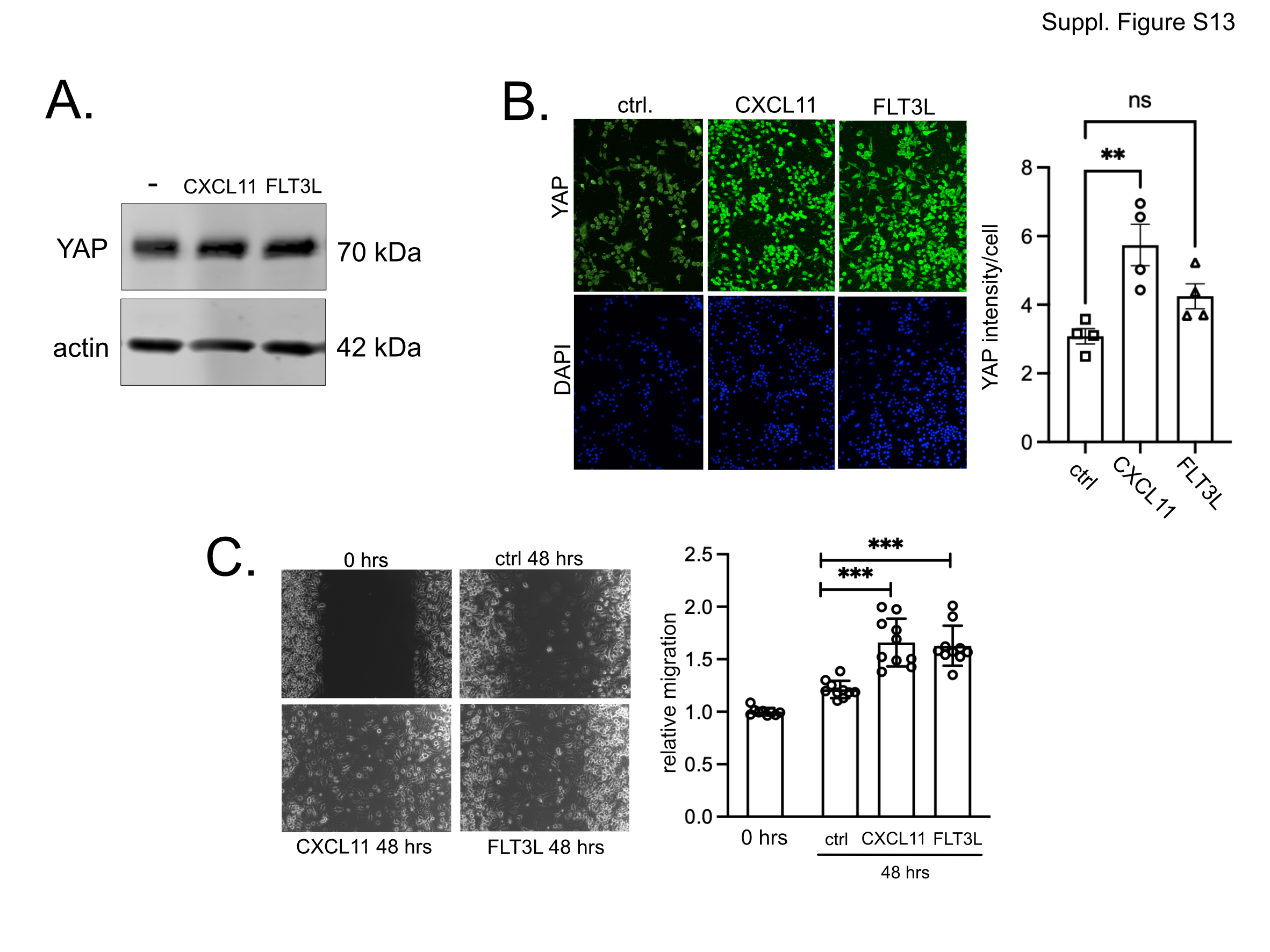

Supplement: Supplementary file 13 — Supplementary file13 (TIFF 1655 KB) Supplementary Figure S13: Effects of CXCL11 and FLT3L on cells of myeloid and endothelial origin. (A.)Western immunoblot of protein fractions isolated from the endothelial cell line SVEC4-10 after treatment with CXCL11 and FLT3L. Signal quantification and normalization with actin showed a 10-20% induction of YAP after FLT3L and CXCL11 treatment, respectively. (B.) Immunofluorescence analysis of myeloid RAW264 cells after CXCL11 and FLT3L treatment demonstrated elevated YAP levels. YAP intensity and the cell number were quantitatively measured. (C.) Lateral migration of an immortalized murine KC cell line. Cells were treated with CXCL11 or FLT3L for 48 hrs. Gaps were digitally documented and cell-free areas were quantified. Nine (0 hrs) and ten (ctrl, CXCL11, and FLT3L after 48 hrs) images were analyzed. Statistical test: ANOVA with Dunnett’s multiple comparisons test. **p≤0.01, ***p≤0.001, ns: not significant. ctrl: control. [file 18_2024_5126_MOESM13_ESM.tiff]
