# Supplementary material for: Dynamic YAP expression in the non-parenchymal liver cell compartment controls heterologous cell communication
Source: Cell Mol Life Sci. 2024 Mar 4;81(1):115. doi: 10.1007/s00018-024-05126-1 (PMC10912141; doi:10.1007/s00018-024-05126-1)
Supplement: Supplementary file 14 — Supplementary file14 (DOCX 19 KB): Supplementary Tables S1 and S2 [file 18_2024_5126_MOESM14_ESM.docx]

**Supplementary Table 1: Primers**

| Primer name | application (ref. seq.) | Sequence (5'-3') |
| --- | --- | --- |
| YAP-for (flox and KO) | genotyping floxed and KO | ACATGTAGGTCTGCATGCCAGAGGAGG |
| YAP-rev1 | genotyping floxed | AGGCTGAGACAGGAGGATCTCTGTGAG |
| YAP-rev2 | genotyping KO | TGGTTGAGACAGCGTGCACTATGGAGC |
| TAZ-for (flox and KO) | genotyping floxed and KO | GGCTTGTGACAAAGAACCTGGGGCTATCTGAG |
| TAZ-rev1 | genotyping floxed | CCCACAGTTAAATGCTTCTCCCAAGACTGGG |
| TAZ-rev2 | genotyping KO | AACTGCTAACGTCTCCTGCCCCTGACCTCTC |
| Cre-for | genotyping Cre | GCCTGCATTACCGGTCGATGCAACGA |
| Cre-rev | genotyping Cre | GTGGCAGATGGCGCGGCAACACCATT |
| YAP-for | qPCR (NC_000075.6) | CAGACGCTGATGAATTCTGCCT |
| YAP-rev | qPCR (NC_000075.6) | CTTGCTCCCATCCATCAGGAA |
| TAZ-for | qPCR (NC_000069.6) | CAGCCGAATCTCGCAATGAATCAC |
| TAZ-rev | qPCR (NC_000069.6) | ACTCATGAGCCCTGTGGGTT |
| tubulin-for | qPCR (NC_000068.7) | TCACTGTGCCTGAACTTACC |
| tubulin-rev | qPCR (NC_000068.7) | GGAACATAGCCGTAAACTGC |
| GAPDH-for | qPCR (NC_000072.6) | TGTCCGTCGTGGATCTGAC |
| GAPDH-rev | qPCR (NC_000072.6) | CCTGCTTCACCACCTTCTTG |
| PPIA-for | qPCR (NC_000077.6) | AGCTGTCCACAGTCGGAAAT |
| PPIA-rev | qPCR (NC_000077.6) | GCATACAGGTCCTGGCATCT |
| TBP-for | qPCR (NC_000083.6) | TTGTCTGCCATGTTCTCCTG |
| TBP-rev | qPCR (NC_000083.6) | CAGGGTGATTTCAGTGCAGA |
| CTGF-for | ChIP qPCR (NC_000076.7) | SimpleChIP® Human CTGF Promoter Primers #14927 |
| CTGF-rev | ChIP qPCR (NC_000076.7) | SimpleChIP® Human CTGF Promoter Primers #14927 |
| Negative Control CTGF-for | ChIP qPCR (NC_000076.7) | SimpleChIP® Human CTGF Upstream Primers #14928 |
| Negative control CTGF-rev | ChIP qPCR (NC_000076.7) | SimpleChIP® Human CTGF Upstream Primers #14928 |
| ICAM1-for | ChIP qPCR (NC_000019.10) | CCGCCCGATTGCTTTAGCTTG |
| ICAM1-rev | ChIP qPCR (NC_000019.10) | GCATTTGTTCCGGAGGGGA |
| Negative Control 1 ICAM1-for | ChIP qPCR (NC_000019.10) | ACTTGCCTGGAACTTATTTCCCT |
| Negative Control 1 ICAM1-rev | ChIP qPCR (NC_000019.10) | TTCAGCAGAGATCTAAATGACGAGA |

**Supplementary Table 2: Antibodies**

| Antibody (clone) | Application, dilution | Source/Company | Cat. No (#); RRID identifier |
| --- | --- | --- | --- |
| β-Actin (13E5) | WB, 1:10,000 | Cell Signaling Technology, Leiden, Netherlands | #4970; AB_2223172 |
| YAP (D8H1X) | IHC/IF, 1:200  coIP, 1:1,000 | Cell Signaling Technology | #14074; AB_2650491 |
| YAP | WB, 1:500 | Cell Signaling Technology | #4912; AB_2218911 |
| TAZ | IHC, 1:250 | Bioss Antibodies Inc. Woburn, MA, USA | #bs-12367R; N/A |
| TAZ | WB, 1:500 | Cell Signaling Technology | #4883; AB_1904158 |
| TAZ | coIP, 1:1,000 | Cell Signaling Technology | #72804; AB_2904134 |
| pan-TEAD | coIP, 1:1,000 | Cell Signaling Technology | #13295S; AB_2687902 |
| CTGF | IHC, 1:200 | Abcam, Berlin, Germany | #6992: AB_305688 |
| F4/80 (BM8) | IHC, 1:250 | BMA Biomedicals, Augst, Switzerland | #T-2006; AB_1227368 |
| CLEC4F | IF, 1:2,000 | R&D Systems, Minneapolis, MN, USA | #AF2784; AB_2081339 |
| CD8 (4SM15) | IHC, 1:200 | Thermo Fisher Scientific | #AB14-0808-82; AB_2572771 |
| Pan-CK | IHC, 1:400 | Agilent, Santa Clara, CA, USA | #Z0622; AB_2650434 |
| MHCII (M5/114.15.2) | IHC, 1:500 | Bio-Techne GmbH, Wiesbaden, Germany | #NBP1-43312; AB_10006678 |
| CD68 | IHC, 1:200 | Abcam | #ab125212; AB_10975465 |
| MPO | IHC, 1:50 | Abcam | #ab9535; AB_307322 |
| B220 (RA3-6B2) | IHC, 1:3,000 | BD Pharmingen, Heidelberg, Germany | #553084; AB_394614 |
| CD3 (SP7) | IHC, 1:500 | Thermo Fisher Scientific | #MA1-90582; AB_1956722 |
| CD4 (4SM95) | IHC, 1:500 | Thermo Fisher Scientific | #14-9766-95; AB_2865432 |
| anti-rabbit IgG Alexa Fluor 488 | IF; 1:250 | Abcam | #150077; AB_2630356 |
